# Towards Explainable Computational Toxicology: Linking Antitargets to Rodent Acute Toxicity

**DOI:** 10.3390/pharmaceutics17121573

**Published:** 2025-12-05

**Authors:** Ilia Nikitin, Igor Morgunov, Victor Safronov, Anna Kalyuzhnaya, Maxim Fedorov

**Affiliations:** 1 Institute for Information Transmission Problems of the Russian Academy of Sciences (Kharkevich Institute), Bolshoy Karetny per. 19, Moscow 127051, Russia; nikitin@iitp.ru (I.N.);; 2 Department of Chemistry, Lomonosov Moscow State University, Leninskie Gory 1/3, Moscow 119991, Russia; 3 Faculty of Applied Mathematics and Computing Sciences, Moscow Institute of Physics and Technology, 9 Institutskiy per., Dolgoprudny 141701, Russia; 4 AI Institute, ITMO University, 49 Kronverksky Pr., St. Petersburg 197101, Russia

**Keywords:** computational toxicology, off-targets, antitargets, toxicodynamics, acute toxicity, LD_50_, molecular docking, inverse docking, safety pharmacology panel, molecular initiating event (MIE)

## Abstract

**Objectives:** One of the major trends in modern computational toxicology is the development of explainable predictive tools. However, the complex nature of the mechanistic representation of biological organisms and the lack of relevant data remain limiting factors. **Methods:** This work provides a publicly available dataset of 12,654 compounds with mouse intravenous ${\mathrm{LD}}_{50}$ values, as well as docking scores (Vina-GPU 2.0) against 44 toxicity-associated proteins. NIH and Brenk filters were applied to refine the chemical space. **Results:** Across the entire protein panel, the human ether-a-go-go–related gene channel (hERG/KCNH2), vasopressin receptor 1A (AVPR1A), the L-type voltage-gated calcium channel Cav1.2 (CACNA1C), the potassium voltage-gated channel subfamily KQT member 1 (KCNQ1) and endothelin receptor A (EDNRA) showed the strongest association with acute toxicity. Statistically significant differences were found in the distribution of ${\mathrm{LD}}_{50}$ values for compounds that bind antitargets compared with non-binders. Using known bioactive molecules such as anisodamine, butaperazine, soman, and several cannabinoids as examples confirmed the effectiveness of inverse docking for elucidating mechanism of action. **Conclusions:** The dataset offers a resource to advance transparent, mechanism-aware toxicity modeling. The data is openly available.

## 1. Introduction

Toxicological profiling of small molecules remains a key task in drug discovery, environmental safety assessment, and regulatory compliance for industrial chemicals [1,2,3]. Traditional experimental approaches are costly, time-consuming, and ethically controversial due to reliance on animal testing [4]. Regulatory frameworks emphasize the need for alternatives. Since the adoption of the European Union’s REACH regulation (Registration, Evaluation, Authorization and Restriction of Chemicals) in 2006 (EU, 2007), the use of QSAR models has become a central computational approach for chemical safety assessment and regulatory decision support [5,6]. Computational toxicology is rapidly gaining importance amid regulatory and ethical demands for faster and more scalable risk assessments. Several initiatives have been launched to develop in silico risk assessment tools. The Next-Generation Risk Assessment approach was initiated and institutionalized under the US EPA as the NexGen program, organized as an interagency collaboration with NIH/NTP, CDC/ATSDR, FDA, and other partners to introduce “next generation” data and tools into risk assessment practices [7,8]. The FDA announced the “New Approach Methodologies” (NAMs) initiative to reduce the number of animals needed for preclinical testing [9]. NAMs methods include in vitro human cell-based systems, such as “organs-on-a-chip,” as well as in silico techniques, including machine learning-based approaches. AI technologies are actively being implemented not only in toxicity prediction but also in other stages of the drug life cycle [10].

Prediction of acute toxicity in particular deserves special attention. It is determined during preclinical drug testing and forms the basis of hazard classifications by the Globally Harmonized System [11] and the US EPA [12]. This trend is also reflected in community efforts, including the 2021 Collaborative Acute Toxicity Modeling Suite (CATMoS) challenge organized by the US National Toxicology Program with participation of 35 international teams [13], as well as the 2023 Syntelly hackathon involving 80 teams [14].

Although in silico methods such as Quantitative Structure–Activity Relationship (QSAR) modeling are gaining popularity, they face significant limitations. Many state-of-the-art approaches rely on chemical descriptors, primarily fingerprints (e.g., MACCS keys, ECFP4)—bit strings indicating the presence or absence of substructures [13,14]. Fingerprints provide limited interpretability: they can highlight the contribution of structural fragments to toxicity, but there is a growing requirement to identify so-called molecular initiating events (MIEs), defined as the first, direct interaction between a chemical or other stressor and a biological molecule, such as a receptor, triggering a cascade of biological events leading to an adverse outcome [15,16]. Therefore, it is necessary to incorporate biological descriptors describing the biological system on which the toxicity endpoint was measured into QSAR models. The development of such interpretable tools in computational toxicology is a cornerstone at present [17]. This is also an essential aspect of the validation of QSAR models, being enshrined in the fifth OECD principle, which implies a QSAR should be associated with “mechanistic interpretation, if possible” [18,19].

Biological descriptors that are gaining popularity in computational toxicology include vector representations of ligand affinities toward a panel of biological targets. The methods used to predict these affinities vary and may include SAR/QSAR modeling, molecular docking, pharmacophore search, and other approaches. Additionally, the qualitative and quantitative composition of the target panel depends on the specific study. These descriptors are also referred to by various names, such as protein target descriptors [20], affinity fingerprints [21], off-target representation [22]. This vector representation of ligand affinities across toxicity-associated proteins may also be referred to as an antitarget interaction profile [23]. Nevertheless, the potential of such representations has been illustrated in several toxicological tasks. For example, J.Liu and colleagues demonstrated the significant potential of an Off-target representation consisting of 242 multi-species proteins (90 unique) in predicting clinical toxicity [22]. The model based on the off-target representation outperformed a similar ECFP4-based model as well as contemporary models like DTox and STDNN-SE [22]. The informativeness of off-target profiles has also been shown in predicting drug-induced liver injury (DILI) [24] and drug-induced kidney injury (DIKI) [25]. At the same time, there are only a few studies implementing antitarget interaction profiles for the prediction of acute toxicity. In 2019, C.H.G. Allen and coauthors published a study on combining Tox21, molecular, and protein target descriptors for an ${\mathrm{LD}}_{50}$ oral dataset with known GHS hazard class labels [20]. Protein target descriptors in this study were represented as a vector of probabilities of interactions with 109 human targets. The results showed that molecular descriptors achieved the highest predictive accuracy. At the same time, the addition of protein target descriptors did not improve model quality. However, we believe that the informativeness of such representations can be enhanced by a set of actions, which are discussed in detail in the present article.

In this work, we performed molecular docking of an ${\mathrm{LD}}_{50}$ (mouse intravenous) ligand dataset to 44 proteins from an unsafe off-target panel [26]. We then demonstrated the impact of antitarget binding on increased ligand toxicity, highlighting the proteins most highly associated with ${\mathrm{LD}}_{50}$. In addition, we show that the raw data require further post-processing. To improve the informativeness of docking scores, it is important to strictly limit the relevant chemical space. In this study, we illustrate the utility of medicinal chemistry filters. The work also discusses intricate correlations between affinities and ${\mathrm{LD}}_{50}$, which may sometimes be misinterpreted. This serves as a sobering reminder on the path toward explainable computational toxicology.

## 2. Materials and Methods

### 2.1. Dataset Preprocessing

A dataset of small molecules with experimental ${\mathrm{LD}}_{50}$ values measured in mice via the intravenous route was obtained from the git repository [27]. We removed chiral molecules with incomplete stereochemical information, as well as those with unspecified E,Z configurations where relevant. We then removed molecules that failed to convert from SMILES to PDBQT format. After these exclusions, the final dataset comprised 12,654 molecules.

### 2.2. Physicochemical Properties Analysis

The number of hydrogen bond donors (HBD), the number of hydrogen bond acceptors (HBA), the octanol–water partition coefficient (logP), the molecular weight (MW), the number of rotatable bonds (RB), and the topological polar surface area (TPSA) were calculated using RDKit 2025.03.04 for all molecules in the dataset [28].

### 2.3. Chemical Space Visualization

To visualize the chemical space of the dataset, the ChemPlot Python library (v. 1.3.1.) was employed, which facilitates dimensionality reduction and plotting of molecular structures based on SMILES representations [29]. Specifically, t-distributed Stochastic Neighbor Embedding (t-SNE) was applied with tailored similarity metrics, incorporating the ${\mathrm{pLD}}_{50}$ (mol/kg) values as the target property for regression analysis.

### 2.4. Molecular Docking

#### 2.4.1. Ligand Preparation

The complete ligand preparation workflow for docking was carried out using the Open Babel v3.1.0 software package [30]. Initially, SMILES structures from the dataset were converted to 2D geometries in SDF format. Three-dimensional geometries were then generated and further optimized using the MMFF94s force field [31], also in SDF format. Next, the structures were converted from SDF to MOL2 format, taking into account protonation at physiological pH 7.4. In the final stage, PDBQT files suitable for docking were generated, with partial charges calculated by the Gasteiger method [32]. A final visual inspection was performed for selected ligands to ensure the correctness of the generated molecular structures, proper representation of chiral centers, and overall structural patterns.

#### 2.4.2. Target Preparation

The full-length three-dimensional structures of 43 biological targets were obtained from the Protein Data Bank (PDB) [33]. For the vasopressin receptor V1A, which is not available in the PDB, a full-length model X5D2B0 built by homology modeling was used [34]. When multiple alternative structures for a given protein were available, preference was given to those previously used in similar molecular docking studies, as well as structures with optimal quality parameters as indicated in the PDB (e.g., Rfree, clashscore, etc.).

Initial target preparation and visualization were performed with PyMOL v3.1.3 [35]. This included the removal of solvent molecules (water) and crystallization artifacts such as organic and inorganic compounds. In addition, ligands in the binding sites and external cofactors were also removed to generate an apo structure of the target. Next, amino acid residues of the orthosteric binding sites were identified, and the coordinates of the center and size of the docking search space (grid box) were determined (Table S1). After preliminary cleaning and definition of the docking search space, biological targets were converted to PDBQT format using the Meeko package [36], which included Gasteiger partial charge calculation and protonation. In cases where residue structures had alternative positions, the most probable conformers (with index A) were always chosen. Further details on PDB-codes, target names, and docking grid boxes can be found in Table S1.

#### 2.4.3. Molecular Docking Software

Molecular docking was performed using the AutoDock Vina scoring function, in a version optimized for GPU to enable faster calculations (Vina-GPU 2.0) [37]. According to recent comparative studies, this scoring function is capable of accurately reproducing ligand poses in binding sites and calculating the docking score [38]. Positive docking scores were replaced with zero values for data denoising.

### 2.5. Orthologous Protein Sequence Alignment

Since the target protein structures were obtained from the PDB for *Homo sapiens*, while the ${\mathrm{LD}}_{50}$ data were measured in mice, we performed pairwise alignment of the 44 target protein sequences between the corresponding human and mouse proteins. This step ensured that these proteins do not differ significantly in their amino acid sequences, validating the comparison of docking scores calculated for the human proteins with experimental data from mice. Alignments were performed using the BLAST (v. 2.7.0) tool provided by the National Institutes of Health (NIH) [39]. Amino acid sequence identity after alignment ranged from 81% to 99%. Further details on alignment scores can be found in Table S1.

### 2.6. Butina Clustering

The Butina clustering algorithm [40], was applied, which groups molecules by identifying common structural patterns and merging them according to molecular similarity assessed by the Tanimoto coefficient. The algorithm implementation involved generating ECFP4 molecular fingerprints for all compounds in the dataset, followed by calculations using bit strings. The similarity threshold for cluster formation by the Tanimoto coefficient was set at 0.65. The dataset comprised 12,654 molecules and was divided into 9665 clusters, indicating a high degree of structural diversity among the compounds. The largest cluster contained 34 molecules, whereas the smallest clusters (singletons) consisted of a single chemical structure, totaling 8326 such cases.

### 2.7. Statistical Analysis

To evaluate the associations between antitarget binding and acute toxicity, two statistical methods were employed.

#### 2.7.1. Mann–Whitney U-Test

Statistical evaluation of differences in toxicity between ligand subsets was performed using the Mann–Whitney U test. The test was applied to compare ${\mathrm{pLD}}_{50}$ distributions between ligands classified as binders (those with docking scores below the predefined threshold of −7 kcal/mol for at least one target, indicating strong binding) and non-binders (those with docking scores above this threshold for all targets) across the entire dataset, as well as for subsets obtained after applying the NIH and Brenk filters. A threshold of *p* < 0.05 was considered statistically significant, indicating that the distributions of ${\mathrm{pLD}}_{50}$ values differ between the two groups.

#### 2.7.2. Spearman Correlations

The nonparametric Spearman rank correlation coefficient was used to quantify the association between docking scores and ${\mathrm{pLD}}_{50}$ (mol/kg) values. Spearman’s correlation measures the degree of monotonic dependence between two variables and does not assume linearity or normality of distributions. For each protein target, Spearman coefficients were computed between docking scores and ${\mathrm{pLD}}_{50}$ values across all molecules in the dataset. Analyses were repeated within major structural clusters identified by Butina clustering.

## 3. Results

### 3.1. Data Overview

**Figure 1:**
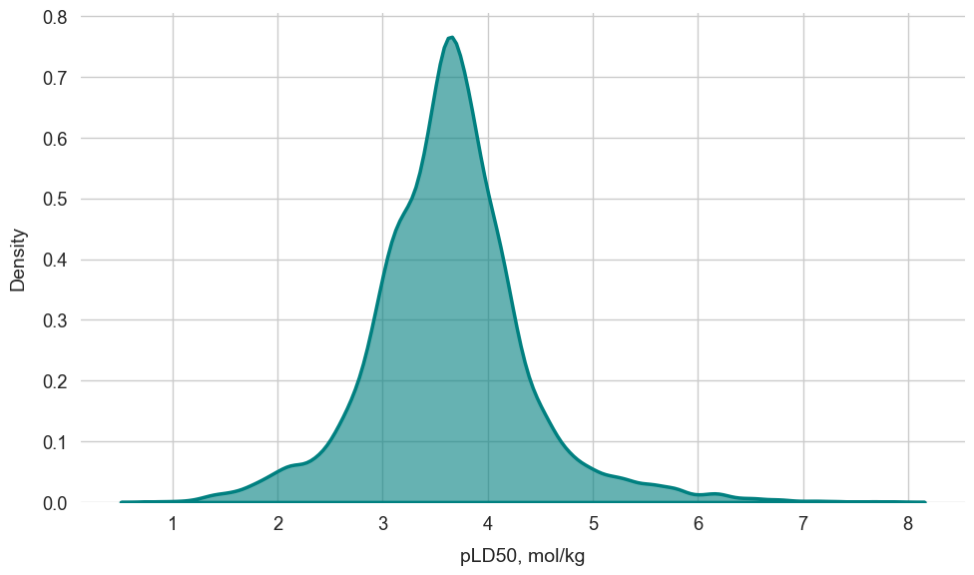
Kernel density estimation of ${\mathrm{pLD}}_{50}$ values (mol/kg) for all compounds in the dataset.

The intravenous route of administration was deliberately selected for the dataset, as it is characterized by 100% bioavailability. We consider this to be the most relevant case for testing the hypothesis regarding the relationship between protein binding affinity and ${\mathrm{LD}}_{50}$, since absorption effects into the systemic circulation are excluded. Mouse was chosen as the animal model due to the highest number of available records. The dataset contains 556,776 docking scores for 12,654 ligands screened against a panel of 44 proteins. The ${\mathrm{pLD}}_{50}$ (mol/kg) values for the molecules range from 0.77 to 7.89, thus encompassing both highly toxic compounds (cannabinoids, organophosphates, cardiac glycosides, etc.) and low-toxicity compounds (sugars, amino acids, etc.). A kernel density plot for ${\mathrm{pLD}}_{50}$ (mol/kg) is shown in Figure 1.

#### 3.1.1. Physicochemical Properties Analysis

**Figure 2:**
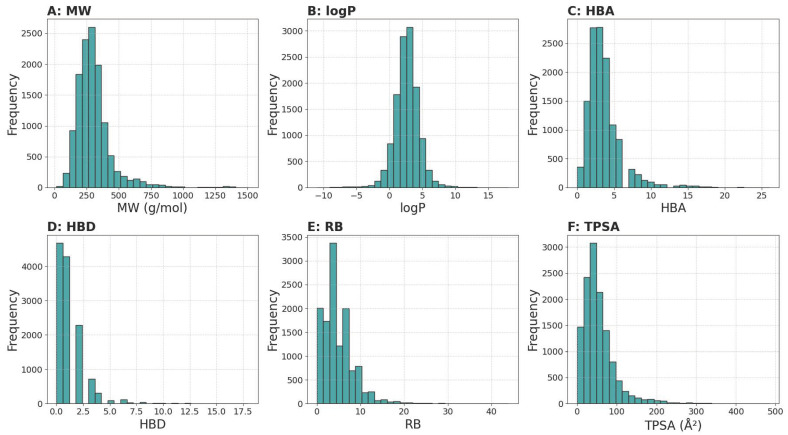
Distribution of physicochemical property values for compounds in the dataset. (**A**) Molecular weight (MW, g/mol); (**B**) Octanol–water partition coefficient (logP, dimensionless); (**C**) Number of hydrogen bond acceptors (HBA, count); (**D**) Number of hydrogen bond donors (HBD, count); (**E**) Number of rotatable bonds (RB, count); (**F**) Topological polar surface area (TPSA, Å^2^). Each panel shows the histogram of the corresponding property for all molecules in the dataset.

The six physicochemical properties of the dataset were analyzed resulting in Figure 2. The hydrogen-bond donor (HBD) count has a mean of 1.19 (SD 1.55) and ranges from 0 to 18; 75% of compounds have ≤2 donors and half have only 1 donor. Hydrogen-bond acceptors (HBA) average 3.57 (SD 2.52) with values from 0 up to 26; most (75%) fall at or below 4 acceptors and the median is 3. Rotatable bonds (RB) show a mean of 4.78 (SD 3.85) spanning 0–44, with 50% of molecules having between 2 and 7 rotatable bonds. Topological polar surface area (TPSA) varies widely from 0 to 483.75 Å^2^ (mean 54.17, SD 45.03), but 50% of compounds lie in the 28.26–67.59 Å^2^ window. Overall, the majority of molecules cluster around low HBD (0–2), moderate HBA (0–4), RB (2–7), and TPSA (28–68 Å^2^).

#### 3.1.2. Visualisation of the Dataset’s Chemical Space

**Figure 3:**
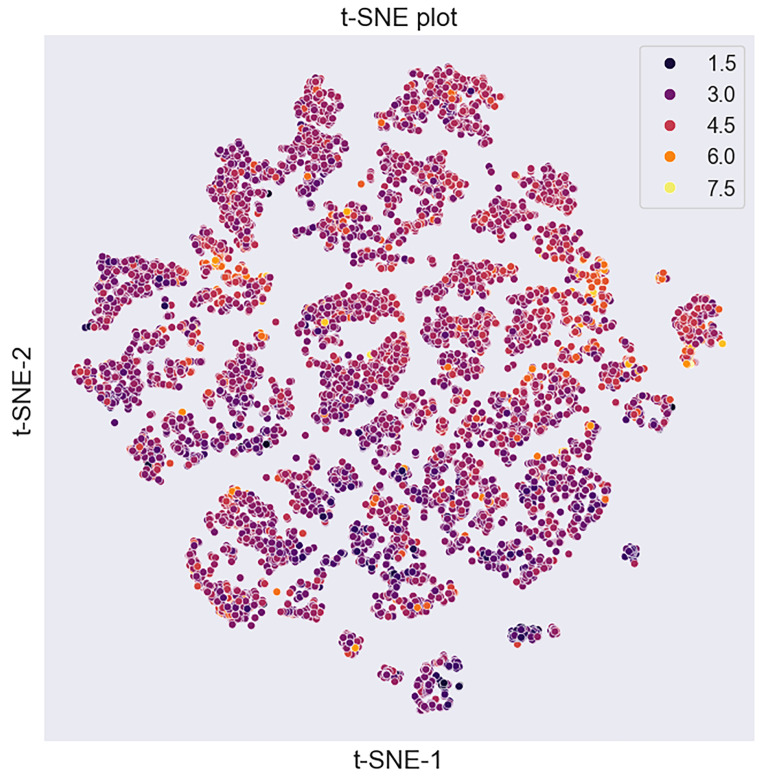
t-SNE visualization of the chemical space of dataset compounds colored by ${\mathrm{pLD}}_{50}$ values (mol/kg), where yellow indicates compounds with the highest toxicity (higher ${\mathrm{pLD}}_{50}$ values), while black corresponds to less toxic compounds (lowest ${\mathrm{pLD}}_{50}$ values).

The chemical space of the dataset was visualized using t-SNE dimensionality reduction. The resulting plot (Figure 3) demonstrates a broad dispersion of points across the reduced 2D space, indicating high chemical diversity within the dataset.

### 3.2. Protein Affinity Profiles

**Figure 4:**
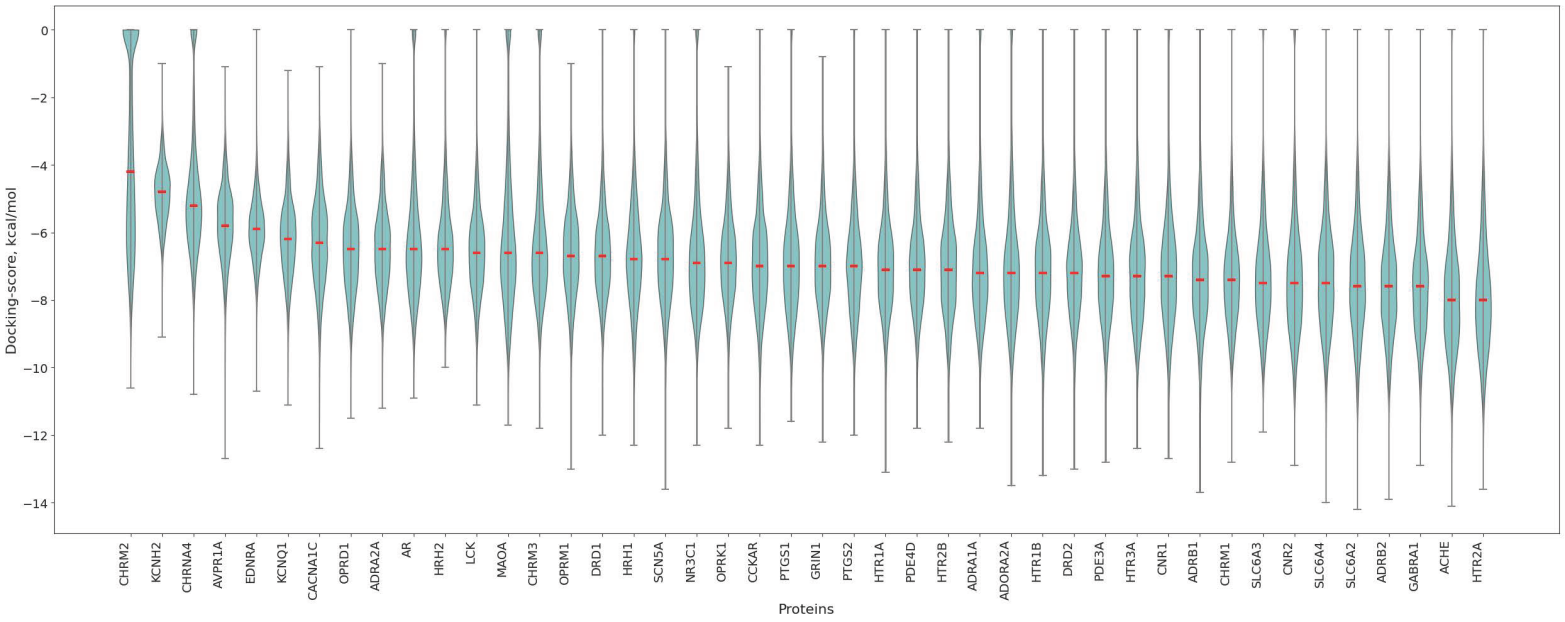
Violin plots of ligand docking scores (kcal/mol) for individual proteins. Median values are highlighted with red lines. Violin plots are ordered from left to right in decreasing order of median docking score. Violin plots illustrate the data distribution by combining a box plot (showing quartiles and median) with a kernel density estimation (where wider sections indicate higher data density, revealing the probability density of values). The embedded box plot shows the median as the central line, the first and third quartiles as the box boundaries (encompassing the interquartile range), whiskers extending to the furthest points within 1.5 times the interquartile range.

After performing molecular docking, it is possible to analyze the distribution of ligand affinities for each protein individually. The corresponding boxplots are shown in Figure 4. It is evident that the protein CHRM2, the M2 muscarinic acetylcholine receptor, displays an anomalous distribution with a median around −4, which is the highest value observed in the entire panel. This result is likely due to the small volume of the protein’s active site. Nearly all medians fall within the range of −6 to −8 kcal/mol, corresponding to fairly strong binding.

### 3.3. Association of Antitargets with ${\mathrm{LD}}_{50}$

**Figure 5:**
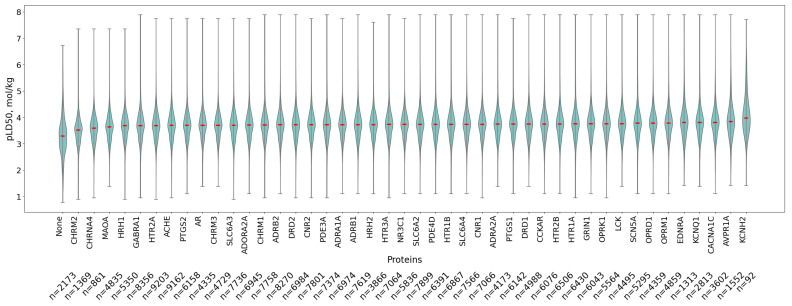
Violin plot distributions of ${\mathrm{pLD}}_{50}$ (mol/kg) values for ligand subsets defined by strong binding (docking score < −7 kcal/mol) to individual protein targets. The “None” category corresponds to ligands not binding to any protein in the panel. Violin plots illustrate the data distribution by combining a box plot (showing quartiles and median) with a kernel density estimation (where wider sections indicate higher data density, revealing the probability density of values). The embedded box plot shows the median as the central red line, the first and third quartiles as the box boundaries (encompassing the interquartile range), whiskers extending to the furthest points within 1.5 times the interquartile range. The sample sizes for each group are indicated below each violin plot.

This study utilizes a panel of proteins proposed by pharmaceutical companies to reduce the risks of acute toxic reactions during drug discovery [26]. However, the relationship of these proteins with ${\mathrm{LD}}_{50}$ in rodents has not been previously discussed. Moreover, it is clear that the relevance of these targets to this endpoint may vary. To assess this, we created ligand subsets for each protein using a docking score threshold of −7 kcal/mol. It is assumed that a molecule with a docking score lower than this threshold indicates strong binding with the protein [41,42]. For each subset, boxplots of the ${\mathrm{pLD}}_{50}$ (mol/kg) distributions were constructed. The results are presented in Figure 5. Ligands that do not bind to any protein showed the lowest ${\mathrm{pLD}}_{50}$ values, i.e., were the least toxic compared to other subsets. This may serve as supporting evidence for the hypothesis that lack of binding to antitargets correlates with low toxicity, except for nonspecific toxicants.

The subset with the highest average ${\mathrm{pLD}}_{50}$ was observed for the potassium ion channel hERG (*the human Ether-a-go-go-Related Gene*; KCNH2), which is one of the most well-known antitargets [43,44,45]. This protein plays a crucial role in cardiac action potential repolarization. It is known that inhibition of the hERG channel may lead to long QT syndrome (LQTS) and even sudden cardiac death [46]. The next strongest association was seen for the vasopressin receptor 1A (AVPR1A), which mediates vasoconstriction and platelet aggregation [26]. This was followed by the L-type voltage-gated calcium channel Cav1.2 (CACNA1C), where interaction is consistent with vascular relaxation, reduced blood pressure, a shortened PR interval, and possible QT shortening [26]. Next is the potassium voltage-gated channel KQT-like member 1 (KCNQ1), whose activation effects include long QT syndrome, potential hearing impairment, deafness, and gastrointestinal symptoms [26]. The fifth most significant target is the endothelin receptor A (EDNRA), responsible for increased blood pressure, aldosterone secretion, and osteoblast proliferation [26]. Thus, all five most significant proteins associated with ${\mathrm{LD}}_{50}$ have adverse effects on the cardiovascular system.

### 3.4. Comparative Toxicity Analysis of Binders and Non-Binders Molecules

According to our hypothesis, if a ligand interacts with at least one of the antitargets, it leads to the occurrence of toxic effects. However, the absence of interactions with the targets cannot conclusively imply that the molecule is safe, as the panel does not cover all possible mechanisms of toxicity and is also poorly informative for nonspecific toxicants. Nevertheless, the hypothesis that ligands with affinity to at least one target are more toxic than those that do not bind to any target was tested. Two approaches were considered.

#### 3.4.1. Raw Data

**Figure 6:**
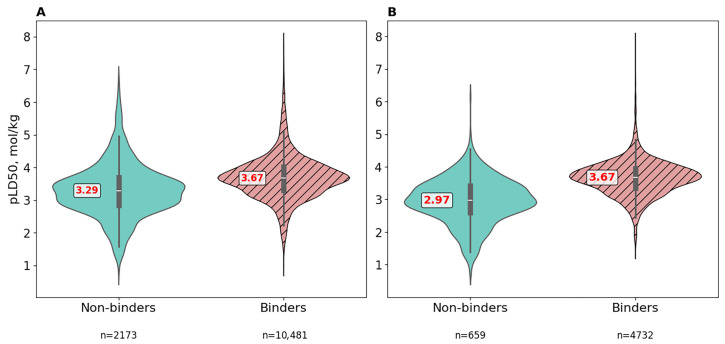
Violin plot distributions of ${\mathrm{pLD}}_{50}$ (mol/kg) values between non-binders (cyan) and binders (pink, hatched) in (**A**) the raw dataset (12,654 compounds) and (**B**) the filtered dataset (5391 compounds) after application of the NIH and Brenk molecular filters. Binders are defined as compounds with at least one docking score below −7 kcal/mol for any protein target. Median values are annotated in red. Violin plots illustrate the data distribution by combining a box plot (showing quartiles and median) with a kernel density estimation (where wider sections indicate higher data density, revealing the probability density of values). The embedded box plot shows the median as the central line, the first and third quartiles as the box boundaries (encompassing the interquartile range), whiskers extending to the furthest points within 1.5 times the interquartile range. The sample sizes for each group are indicated below each violin plot.

Based on the initial dataset, two subsets were formed. The first included ligands that do not bind to any protein (do not exceed the docking score threshold for any target). The second comprised ligands that exceeded the threshold for at least one protein. Boxplots of the corresponding distributions are shown in Figure 6A. The Mann–Whitney statistical test was then conducted to assess differences in ${\mathrm{LD}}_{50}$ distributions between the two subsets. According to the results, the *p*-value was less than 0.05, indicating a statistically significant difference in ${\mathrm{LD}}_{50}$ distributions between the groups. This result is consistent with our hypothesis.

#### 3.4.2. Preprocessed Data Using Molecular Filters

As mentioned above, comparing binders and non-binders using raw data can be misleading. This is because molecules with low affinity for the panel of targets may still be toxic for other reasons. To partially address this problem, we applied molecular filters:

1. NIH filter
  Developed by the National Institutes of Health, these filters flag functional groups and scaffolds associated with pan-assay interference compounds (PAINS), reactive electrophiles, redox-active motifs, and other liabilities [47].
2. Brenk filter
  Proposed by Brenk et al. (2008), this set comprises SMARTS patterns targeting: Toxicophores (e.g., anilines), highly lipophilic aromatic systems prone to promiscuous binding, Reactive functionalities (e.g., epoxides, aziridines) [47,48].

These filters identify toxicophores, which generally exert toxicity unrelated to protein targets. For example, aniline-like amines are metabolized by CYP450 family enzymes to nitrenium ions, which subsequently alkylate DNA [49,50]. Another example includes surfactants such as saponine derivatives included in the Brenk filter. It is known that the toxicity of surfactants is often associated with disruption of cell membrane integrity [51,52,53]. In such cases, even if a molecule does not bind to any protein, it can still be highly toxic.

For the reasons described above, the dataset was filtered using the Brenk and NIH filters, reducing it from 12,654 to 5391 molecules. Violin plots of ${\mathrm{LD}}_{50}$ distributions for the remaining molecules were then constructed (Figure 6B). The difference in medians nearly doubled (from 0.38 to 0.7) compared to the raw data. The distribution for binders remained similar, while the toxicity of non-binders decreased as expected. The Mann–Whitney test again showed a statistically significant difference between distributions (*p* < 0.05).

Thus, this result provides grounds to suggest the existence of a specific region of chemical space for which the concept of toxicity prediction based on antitarget interaction profiles is more relevant.

### 3.5. Confirmation of the Pharmaco- and Toxicodynamics of Known Bioactive Molecules via Inverse Docking

**Figure 7:**
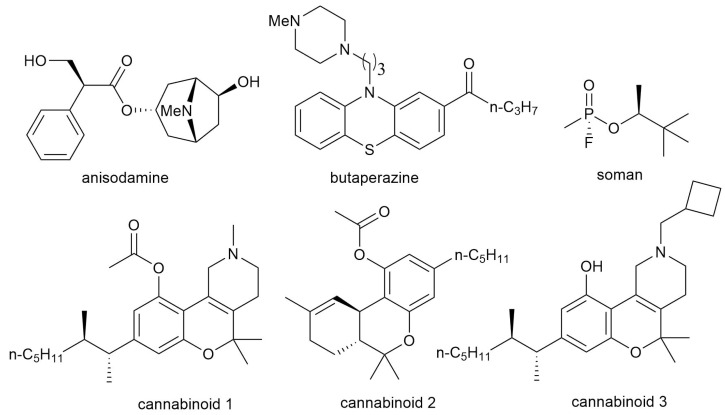
Chemical structures of six compounds from the dataset with well-characterized pharmacodynamic and toxicodynamic mechanisms of action. These are anisodamine, butaperazine, soman and 3 cannabinoids. Cannabinoid 1 is [2,5,5-trimethyl-8-(3-methyloctan-2-yl)-3,4-dihydro-1H-chromeno[4,3-c]pyridin-10-yl] acetate, cannabinoid 2 is (6,6,9-trimethyl-3-pentyl-6a,7,8,10a-tetrahydrobenzo[c]chromen-1-yl) acetate (Tetrahydrocannabinol acetate), cannabinoid 3 is 2-(cyclobutylmethyl)-5,5-dimethyl-8-(3-methyloctan-2-yl)-3,4-dihydro-1H-chromeno[4,3-c]pyridin-10-ol.

Interpretable computational toxicology involves elucidating the mechanisms of toxicity for small molecules. In other words, it aims to identify the so-called molecular initiating event (MIE), which typically refers to an interaction with a specific protein. Various approaches are used to identify such targets, collectively referred to as target fishing [54,55]. Docking ligands to a panel of proteins (inverse docking) is one of these techniques. Accordingly, our dataset can be used for predicting the mechanism of action of a ligand. To illustrate this, let us consider 6 molecules from the dataset with well-characterized pharmaco- and toxicodynamics. Their chemical structures are shown in the Figure 7.

**Figure 8:**
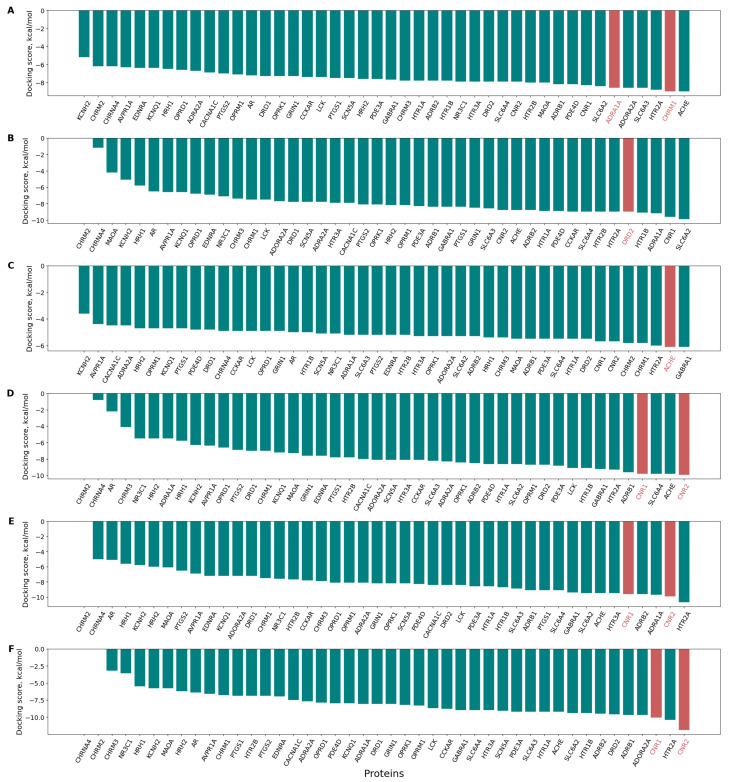
Antitarget interaction profiles of 6 selected compounds against the protein panel. The X-axis represents the 44 proteins sorted by increasing predicted affinity, while the Y-axis shows the docking score in kcal/mol. Docking scores for proteins with unknown interactions are shown as teal bars, while those for proteins with in vitro confirmed ligand-protein interactions are highlighted as red bars. (**A**) Anisodamine: Muscarinic acetylcholine receptor M1 and α−1 adrenergic receptor; (**B**) Butaperazine: Dopamine D2 receptor; (**C**) Soman: Acetylcholinesterase; (**D**–**F**) Cannabinoids 1–3: Cannabinoid receptors 1 and 2 (in vitro confirmed interactions).

The antitarget interaction profiles demonstrate that known targets for the selected molecules exhibit low docking scores, highlighting the dataset’s utility for predicting mechanisms of action. The antitarget interaction profiles with the selected molecules are shown in Figure 8, where the target proteins are highlighted in red. Anisodamine, which interacts with the Muscarinic acetylcholine receptor M1 and the α−1 adrenergic receptor (Figure 8A) [56]. Butaperazine, a phenothiazine derivative that exerts antipsychotic effects through blockade of dopamine D2 receptors (Figure 8B). Soman is the widely known toxicant soman, which inhibits acetylcholinesterase (Figure 8C). Finally, cannabinoids 1–3 interact with Cannabinoid receptors 1 and 2, shown in Figure 8D–F, respectively.

Thus, for several compounds, it is evident that molecular docking enables assessment of their mechanisms of interaction with biological targets. Consequently, our dataset is useful for evaluating mechanisms of action. However, high docking scores are often observed for proteins without confirmed in vitro interactions, indicating potential data noise. The use of consensus affinity evaluation methods, such as incorporating QSAR and pharmacophore modeling, would likely allow for a more precise determination of toxicodynamics.

### 3.6. Correlations Between ${\mathrm{LD}}_{50}$ and Protein Binding Affinity

The informativeness of a feature for predicting a target variable is often evaluated by the degree of statistical dependence between them. In this study, we investigated the relationship between docking scores and ${\mathrm{LD}}_{50}$ values using the nonparametric Spearman correlation coefficient. The analysis was performed on the entire dataset as well as within each structural cluster separately to identify characteristic patterns for different chemical families.

#### 3.6.1. Initial Dataset

**Figure 9:**
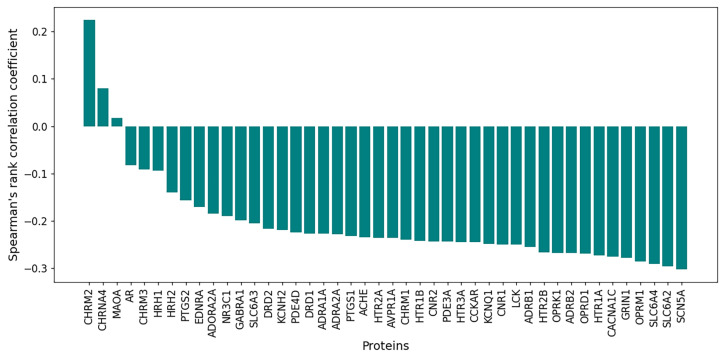
Spearman rank correlation coefficients between docking scores and ${\mathrm{pLD}}_{50}$ (mol/kg) values for 44 protein targets in the raw dataset (12,654 compounds). Negative values indicate that increased binding affinity is associated with greater toxicity.

For each of the 44 proteins, the Spearman correlation coefficient was calculated based on all 12,654 ligands. According to our hypothesis, increased affinity for the proteins (i.e., lower docking scores) leads to greater toxicity, expressed as higher ${\mathrm{pLD}}_{50}$ values. In other words, an inverse relationship between docking score and ${\mathrm{pLD}}_{50}$ is expected, corresponding to negative correlation coefficients. The values ranged from 0.2 to −0.3, with a median of −0.14 (Figure 9), indicating that there is almost no association between affinity and ${\mathrm{LD}}_{50}$ in the raw data. However, this result is expected for at least three reasons. Firstly, some molecules do not bind to the proteins and thereby introduce noise into the analysis; as previously discussed, a molecule may be toxic due to activity on other proteins or for other reasons. Secondly, docking scores below the binding threshold also contribute noise. Thirdly, comparing docking scores for molecules from different regions of chemical space can distort the results. It is therefore more appropriate to analyze chemical clusters separately.

#### 3.6.2. Cluster Analysis

**Figure 10:**
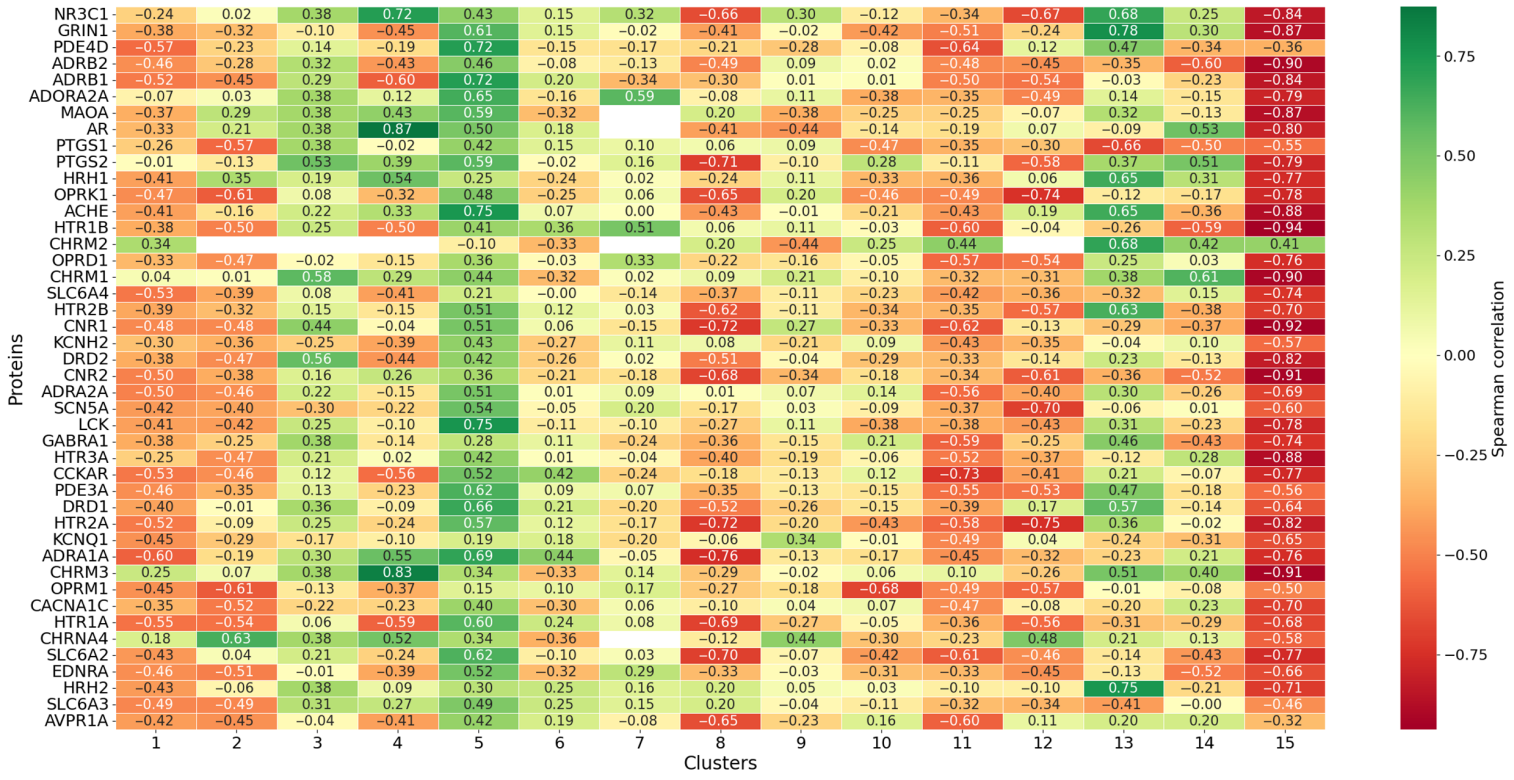
Heatmap showing Spearman correlation coefficients between docking scores and ${\mathrm{pLD}}_{50}$ (mol/kg) values for 44 protein targets across the 15 largest chemical clusters obtained by Butina clustering. Clusters are numbered 1 to 15 (x-axis), proteins (y-axis); missing values indicate absence of binding for all compounds in a cluster for the corresponding protein.

For the reasons mentioned above, it was of particular interest to analyze the correlations between ${\mathrm{pLD}}_{50}$ (mol/kg) and docking scores across different chemical clusters. To this end, Butina clustering was employed. The 15 largest clusters were selected for further analysis, with cluster sizes ranging from 13 to 34 molecules. The chemical structures of these compounds are provided in Supplementary Materials Figures S1–S15. For each of the remaining clusters, 44 Spearman correlation coefficients were calculated (Figure 10). If all compounds in a cluster had a docking score of zero for a particular protein, the corresponding entry in the heatmap is marked as missing. As shown in Figure 10 the correlations vary markedly depending on the cluster. This indicates that raw data indeed require additional post-processing.

**Figure 11:**
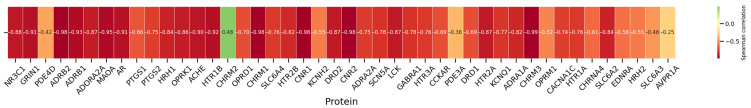
Heatmap of Spearman correlation coefficients between logP values and docking scores for aliphatic carboxylic acids from cluster 15 across the protein panel. Each cell shows the correlation for a given protein.

Particular attention is drawn to cluster 15 due to the presence of strong negative correlations across almost the entire protein panel. Although such values are consistent with our hypothesis, the presence of correlations with so many proteins is suspicious. Typically, toxicants have one or a few specific target proteins. This cluster contains aliphatic carboxylic acids with unbranched chains (Figure S15). These compounds show a high correlation between logP and ${\mathrm{LD}}_{50}$ (Spearman correlation coefficient = 0.92). In this case, it is reasonable to expect that the docking scores contain a hidden variable—logP. To test this hypothesis, we calculated correlations between the logP values of the acid molecules and their affinities for each protein (Figure 11). The results reveal a clear dependence to varying degrees across each protein, indicating an obvious influence of chemical structure homology on the overall docking score. Despite its simplicity, this finding is important for interpretation. Sometimes in the literature, correlations between protein affinity and ${\mathrm{LD}}_{50}$ or other toxicity endpoints are reported [57]. However, using the example of aliphatic carboxylic acids, we have shown that the presence of such correlations alone does not justify conclusions about the toxicant’s mechanism of action.

## 4. Discussion

### Challenges and Prospects

It should be noted that this study is only a step toward explainable computational toxicology. It is important to understand the limitations and assumptions of the method. In this subsection, we outline several points that should be taken into account to improve approaches for predicting toxicity based on affinities for a protein panel.

1. Mechanistic domain
  The panel-based approach is relevant when ligand toxicity is driven by interaction with one of the listed proteins. Therefore, it is crucial to properly filter out nonspecific toxicants. Moreover, the panel of 44 proteins is not exhaustive and requires expansion. Toxicity targets may also include other biopolymers (such as DNA), not just proteins.
2. Binding affinity prediction methods
  In this study, we used molecular docking to assess affinities. However, this method involves several assumptions and limitations [58,59,60,61]. In particular, an accurate three-dimensional structure of the target protein is essential, as it enables the prediction of ligand-protein interactions by modeling binding poses and affinities. However, the selected protein structure may be incorrectly chosen due to factors such as incomplete experimental data or modeling errors. For example, while full-length protein structures are preferable for comprehensive analysis, sometimes only individual domains are available. A promising direction is to use consensus approaches that combine multiple in silico tools (QSAR, pharmacophore search, etc.) for affinity prediction.
3. Structures of orthologous proteins
  One limitation of this study is the reliance on orthologous proteins 3D-structures. This approach may introduce inaccuracies in docking predictions, as subtle structural variations between orthologs—such as differences in active site conformations—could alter ligand binding affinities and lead to misestimation of toxicodynamics. Furthermore, the absence of comprehensive species-specific crystallographic data for many antitarget proteins necessitates this approximation, potentially limiting the generalizability of the findings to real-world interspecies extrapolations in toxicological assessments.
4. Affinity
  Docking scores estimate ligand affinity for proteins, but efficacy is critical for bioactivity. Molecules may have identical docking scores yet exert completely different effects on a protein (agonists, antagonists, inverse agonists) [62,63].
5. Binding site
  Affinity assessments of small molecules for proteins usually focus on the orthosteric site. However, molecules may also bind to allosteric sites, acting as either positive or negative allosteric modulators (PAMs, NAMs) [64].
6. Absorption and bioavailability
  Molecules administered into the body are characterized by bioavailability—the percentage of the substance that reaches the systemic circulation. In our study, we chose the intravenous route of administration, ensuring 100% bioavailability for all compounds [65]. However, applying this concept to other datasets may be challenging. Another important aspect of bioavailability is formulation. Even with the same route of administration and substance, bioavailability may vary significantly depending on formulation. Various methods for increasing bioavailability are known, such as micronization, solid dispersion and so on [66,67]. This issue also relates more broadly to data aggregation. It is important to pay close attention to experimental protocols when compiling datasets.
7. Distribution
  Docking assumes inevitable ligand–protein encounters. However, xenobiotics administered to the body do not necessarily reach every protein, due to distribution. For example, for proteins located in the CNS, a ligand must cross the blood–brain barrier [68]. To enhance the informativeness of binding data in toxicity prediction, information about protein localization should be considered.
8. Metabolism
  In the body, xenobiotics undergo biotransformation reactions that alter their structures, primarily via CYP450 family enzymes [69,70]. A single xenobiotic may yield dozens or even hundreds of metabolites, which can differ markedly in bioactivity. For example, prodrugs are a class whose therapeutic effect depends on the formation of active metabolites. Sofosbuvir is a prodrug that is metabolized to the active antiviral agent GS-461203 (2’-deoxy-2’-α-fluoro-β-C-methyluridine-5’-triphosphate), which acts as a defective substrate for the NS5B protein, a viral RNA polymerase [71]. In our study, docking was performed only for the parent compounds. Explicitly accounting for metabolism is quite challenging, as it requires predicting metabolite structures, which is still subject to uncertainty. Moreover, this would significantly increase the scale of ligand–protein docking calculations.
9. Excretion
  Excretion is a key pharmacokinetic process determining the elimination of xenobiotics and their metabolites from the body through routes such as renal filtration, biliary secretion, or pulmonary exhalation. Variability in excretion rates and pathways can greatly influence systemic exposure and toxicity outcomes due to accumulation [72].
10. Constraints of the model interpretability approach
  As discussed above in Section 3.6.2, a strong correlation may sometimes be misinterpreted. It is important to recognize that in such cases, the observed relationship does not necessarily reflect a true mechanism of action but rather a correlation with other descriptors (such as logP). Moreover, molecular docking can produce false-positive results, which in turn may lead to misleading interpretations of the underlying biological mechanism.

## 5. Conclusions

Linking affinities for antitarget proteins with acute toxicity endpoints represents a promising avenue for developing interpretable predictive tools on the path toward explainable computational toxicology. In this study, we present a publicly available dataset comprising 12,654 compounds with experimentally measured ${\mathrm{LD}}_{50}$ values in mice (intravenous administration) and docking scores against a panel of 44 antitargets. This enables mechanistic insights into acute rodent toxicity. Through comprehensive analyses, we demonstrated that antitarget interaction profiles contribute to ${\mathrm{LD}}_{50}$ prediction, with a marked increase in informativeness achieved by medicinal chemistry-aware post-processing, such as molecular filtering to delineate relevant chemical spaces. Key findings include the identification of antitargets related to the cardiovascular system as significant toxicity factors, statistically significant differences between binders and non-binders, and the validation of known pharmaco- and toxicodynamic mechanisms for selected compounds. While our approach highlights the potential of antitarget-based representations for linking chemical structures to biological outcomes, it also brings attention to challenges. These include the need to expand protein panels, implement consensus affinity prediction methods and account for pharmacokinetic factors. The dataset and methodology presented serve as a fundamental resource for advancing interpretable models in computational toxicology, facilitating safer chemical design and regulatory decisions. Future efforts should focus on integrating physiological and biochemical data to enhance the relevance and informativeness of antitarget interaction.

## Data Availability

The dataset is publicly available at https://github.com/chemagents/ld50-antitargets, accessed on 13 October 2025.

## Supplementary Materials

The following supporting information can be downloaded at: https://www.mdpi.com/article/10.3390/pharmaceutics1010000/s1, Table S1: Protein Target Annotations and Box Parameters. This table provides detailed information about all protein targets used in the study, including target name, PDB code, UniProt ID, organism, percentage sequence identity with the mouse homolog, and docking box dimensions and centers used for molecular docking experiments. Figures S1–S15: Molecular structures of compounds in Butina clusters 1–15. Figure S16: Scatter plots of docking scores versus ${\mathrm{pLD}}_{50}$ (mol/kg) for 12,654 ligands across 44 proteins, sorted by increasing *p*-value of Spearman’s rank correlation. Each panel displays the protein identifier and the corresponding Spearman’s correlation coefficient (ρ).
