# Supplementary material for: Towards Explainable Computational Toxicology: Linking Antitargets to Rodent Acute Toxicity
Source: Pharmaceutics. 2025 Dec 5;17(12):1573. doi: 10.3390/pharmaceutics17121573 (PMC12737167; doi:10.3390/pharmaceutics17121573)
Supplement: Supplementary file 1 [file pharmaceutics-17-01573-s001.zip › pharmaceutics-3955649-supplementary.pdf]

Supporting Information

Table S1. Protein Target Annotations and Box Parameters

Name	PDB код	UNIPROT ID	Organism	Mouse Sequence Align (%)	BOX
Glucocorticoid receptor	1m2z	P04150	Homo sapiens	88.18	Box dimensions (22.48, 16.18, 20.32) Box center (18.16, 24.43, -13.48)
Tyrosine-protein kinase Lck	6pdj	P06239	Homo sapiens	96.86	Box dimensions (23.96, 23.08, 23.99) Box center (0.37, -0.40, -10.09)
Beta-2 adrenergic receptor	2rh1	P07550	Homo sapiens	87.08	Box dimensions (24.17, 22.69, 26.72) Box center (-32.48, 6.09, 8.61)
Muscarinic acetylcholine receptor M2	4mqs	P08172	Homo sapiens	96.35	Box dimensions (17.47, 20.88, 23.16) Box center (-4.52, -11.59, -3.98)
Beta-1 adrenergic receptor	2vt4	P08588	Homo sapiens	87.87	Box dimensions (23.29, 21.72, 18.89) Box center (32.31, 13.97, -32.84)
5-hydroxytryptamine receptor 1A	8pjk	P08908	Homo sapiens	88.36	Box dimensions (18.87, 22.82, 22.42) Box center (162.82, 171.23, 142.88)
Alpha-2A adrenergic receptor	6kux	P08913	Homo sapiens	92.47	Box dimensions (27.82, 25.67, 34.60) Box center (0.56, -7.80, -23.28)
Androgen receptor	3b66	P10275	Homo sapiens	84.29	Box dimensions (17.28, 19.32, 24.43)

					Box center (26.86, 1.17, 9.22)
Muscarinic acetylcholine receptor M1	5cxv	P11229	Homo sapiens	98.91	Box dimensions (18.28, 18.26, 24.30) Box center (-11.12, -13.34, 37.23)
D(2) dopamine receptor	6cm4	P14416	Homo sapiens	95.5	Box dimensions (20.90, 18.74, 27.13) Box center (11.34, 5.41, -8.67)
Gamma-aminobutyric acid receptor subunit alpha-1	6x3x	P14867	Homo sapiens	98.46	Box dimensions (29.14, 18.13, 29.44) Box center (94.37, 124.75, 104.23)
Muscarinic acetylcholine receptor M3	8e9y	P20309	Homo sapiens	91.69	Box dimensions (18.05, 21.99, 16.28) Box center (124.07, 118.82, 101.71)
Amine oxidase [flavin-containing] A	2z5x	P21397	Homo sapiens	88.12	Box dimensions (18.48, 15.04, 16.59) Box center (43.63, 28.56, -15.71)
Cannabinoid receptor 1	5u09	P21554	Homo sapiens	97.04	Box dimensions (16.87, 30.66, 25.08) Box center (22.56, 1.63, -8.40)
D(1A) dopamine receptor	7ljd	P21728	Homo sapiens	91.65	Box dimensions (26.26, 23.51, 26.75) Box center (97.86, 107.62, 74.58)
Acetylcholinesterase	4ey7	P22303	Homo sapiens	88.44	Box dimensions (24.74, 25.80, 24.22) Box center (-12.33, -46.48, 31.16)
Prostaglandin G/H synthase 1	3kk6	P23219	Homo sapiens	89.93	Box dimensions (20.91, 22.91, 29.92) Box center (-22.69,

					51.06, 41.50)
Sodium-dependent noradrenaline transporter	8wty	P23975	Homo sapiens	94.33	Box dimensions (26.82, 29.57, 18.80)<br>Box center (98.48, 110.56, 110.09)
Histamine H2 receptor	8yn3	P25021	Homo sapiens	85.79	Box dimensions (16.63, 19.65, 27.00)<br>Box center (96.25, 112.24, 75.80)
Endothelin-1 receptor	8xvk	P25101	Homo sapiens	92.27	Box dimensions (21.94, 26.89, 30.29)<br>Box center (154.19, 151.54, 169.80)
5-hydroxytryptamine receptor 1B	4iar	P28222	Homo sapiens	92.53	Box dimensions (19.85, 20.57, 24.71)<br>Box center (-11.78, -19.49, 20.00)
5-hydroxytryptamine receptor 2A	7wc9	P28223	Homo sapiens	91.51	Box dimensions (24.06, 25.41, 31.63)<br>Box center (-30.00, -11.88, 143.90)
Adenosine receptor A2a	2ydo	P29274	Homo sapiens	81.0	Box dimensions (18.57, 18.84, 19.90)<br>Box center (-28.69, 8.07, -22.13)
Sodium-dependent serotonin transporter	5i71	P31645	Homo sapiens	92.54	Box dimensions (28.34, 24.43, 20.20)<br>Box center (-39.07, -16.46, 3.04)
Cholecystokinin receptor type A	7f8y	P32238	Homo sapiens	88.99	Box dimensions (24.25, 23.85, 20.26)<br>Box center (9.01, 24.34, 56.06)
Cannabinoid receptor 2	6kpf	P34972	Homo sapiens	82.71	Box dimensions (25.00, 21.21, 21.74)<br>Box center (109.34, 106.59, 125.78)

Alpha-1A adrenergic receptor	7ym8	P35348	Homo sapiens	91.85	Box dimensions (20.19, 17.65, 29.61) Box center (139.86, 143.29, 154.54)
Prostaglandin G/H synthase 2	3ln1	P35354	Homo sapiens	86.75	Box dimensions (25.26, 24.54, 23.92) Box center (32.00, -26.40, -18.73)
Histamine H1 receptor	3rze	P35367	Homo sapiens	77.91	Box dimensions (24.85, 24.84, 30.75) Box center (16.61, 33.13, 17.89)
Mu-type opioid receptor	8ef6	P35372	Homo sapiens	94.0	Box dimensions (24.64, 26.36, 32.34) Box center (102.49, 103.58, 133.41)
Vasopressin V1a receptor	V1A	P37288	Homo sapiens	82.0	Box dimensions (27.50, 23.38, 22.28) Box center (164.16, 120.96, -56.08)
Delta-type opioid receptor	4n6h	P41143	Homo sapiens	93.55	Box dimensions (28.61, 31.55, 40.60) Box center (-3.90, -74.35, 61.32)
Kappa-type opioid receptor	4djh	P41145	Homo sapiens	93.68	Box dimensions (22.48, 23.20, 28.66) Box center (2.64, -22.05, 64.15)
5-hydroxytryptamine receptor 2B	5tvn	P41595	Homo sapiens	83.16	Box dimensions (22.04, 32.47, 25.21) Box center (-20.46, -23.56, 11.12)
Neuronal acetylcholine receptor subunit alpha-4	8st0	P43681	Homo sapiens	83.36	Box dimensions (18.01, 14.34, 16.09) Box center (219.45, 153.57, 175.51)
5-hydroxytryptamine receptor 3A	6y1z	P46098	Homo	83.23	Box dimensions (23.22,

			sapiens		17.51, 21.07)<br>Box center (135.33, 154.91, 159.65)
Potassium voltage-gated channel subfamily KQT member 1	7xnk	P51787	Homo sapiens	88.2	Box dimensions (31.90, 24.34, 24.09)<br>Box center (129.38, 105.93, 100.80)
Sodium-dependent dopamine transporter	9eo4	Q01959	Homo sapiens	93.55	Box dimensions (25.23, 20.98, 21.12)<br>Box center (-39.60, -1.08, 57.44)
Glutamate receptor ionotropic, NMDA 1	1pbq	Q05586	Homo sapiens	99.04	Box dimensions (21.60, 27.64, 22.10)<br>Box center (33.10, 27.84, -44.27)
3',5'-cyclic-AMP phosphodiesterase 4D	1xoq	Q08499	Homo sapiens	98.48	Box dimensions (20.97, 19.58, 25.64)<br>Box center (13.79, 28.53, 54.60)
Voltage-gated inwardly rectifying potassium channel KCNH2	5va1	Q12809	Homo sapiens	95.96	Box dimensions (30, 30, 30)<br>Box center (80.83, 68.74, 82.48)
Voltage-dependent L-type calcium channel subunit alpha-1C	8fhs	Q13936	Homo sapiens	90.96	Box dimensions (20.08, 28.52, 26.27)<br>Box center (150.39, 161.28, 147.83)
cGMP-inhibited 3',5'-cyclic phosphodiesterase 3A	7kwe	Q14432	Homo sapiens	83.58	Box dimensions (18.35, 19.95, 31.78)<br>Box center (-19.25, -20.49, 38.75)
Sodium channel protein type 5 subunit alpha	6lqa	Q14524	Homo sapiens	94.45	Box dimensions (27.66, 31.08, 28.79)<br>Box center (130.16, 125.42, 136.52)

Cluster 1

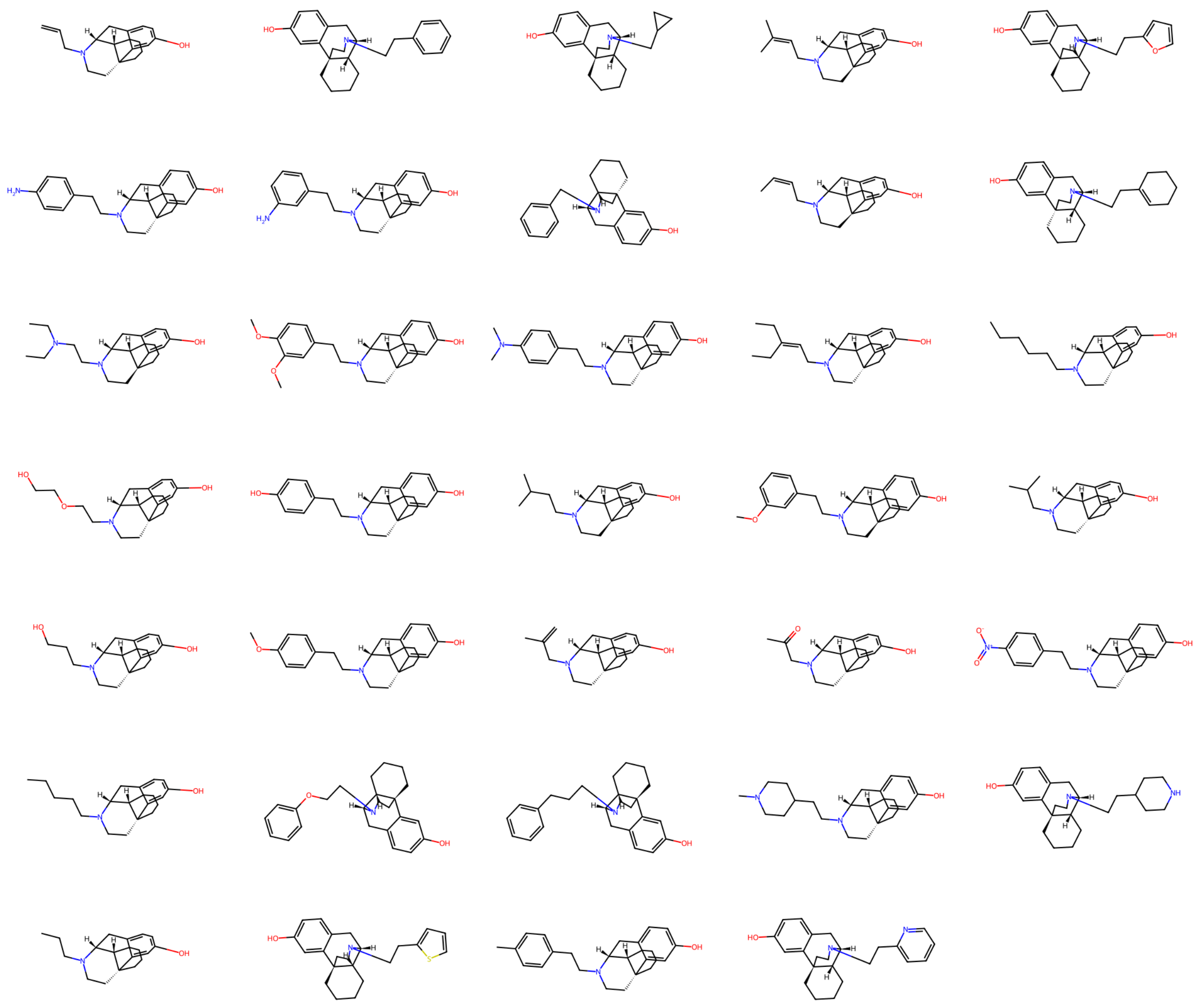

Figure S1. Molecular structures of compounds in cluster 1.

Cluster 2

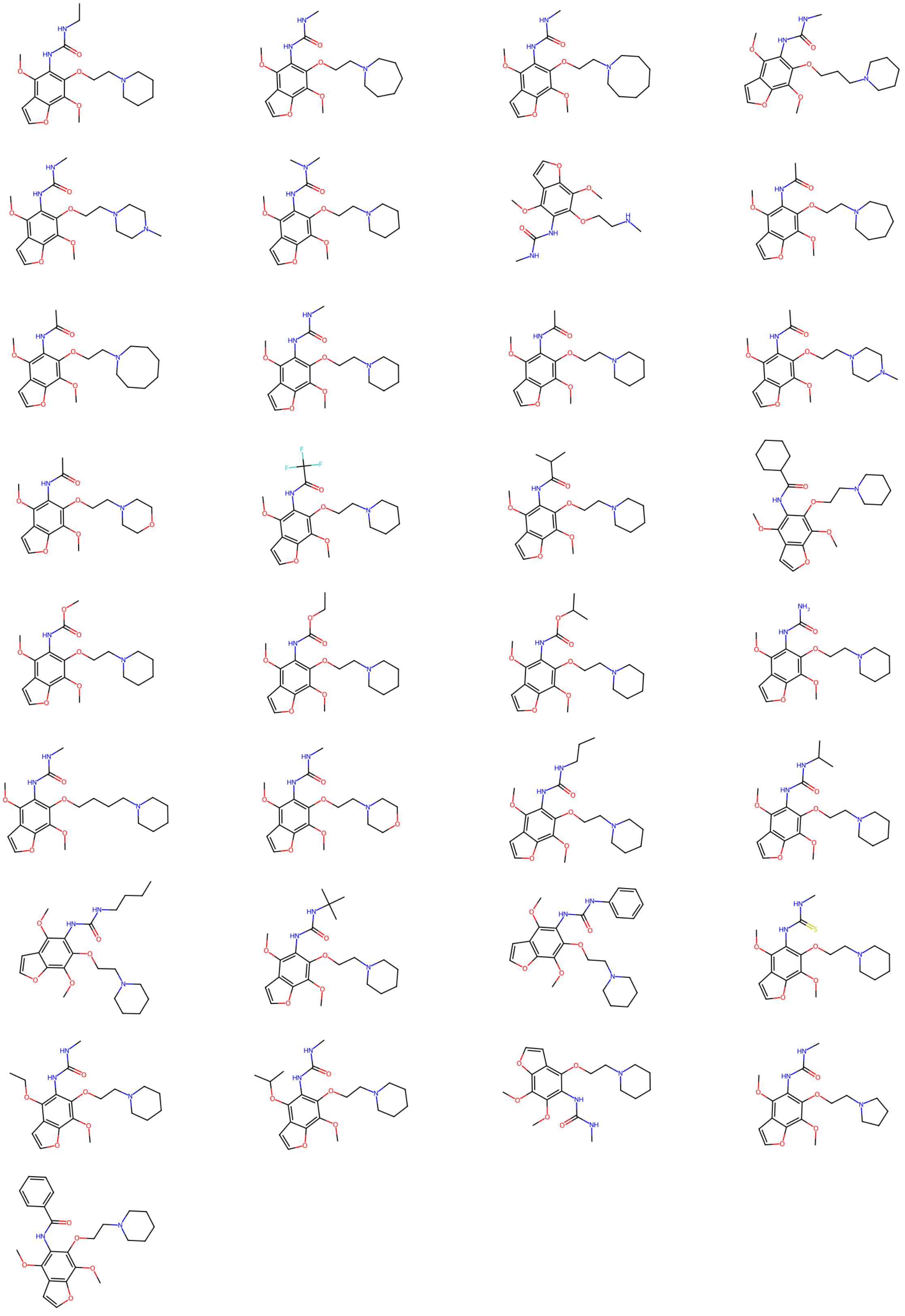

Figure S2. Molecular structures of compounds in cluster 2.

Cluster 3

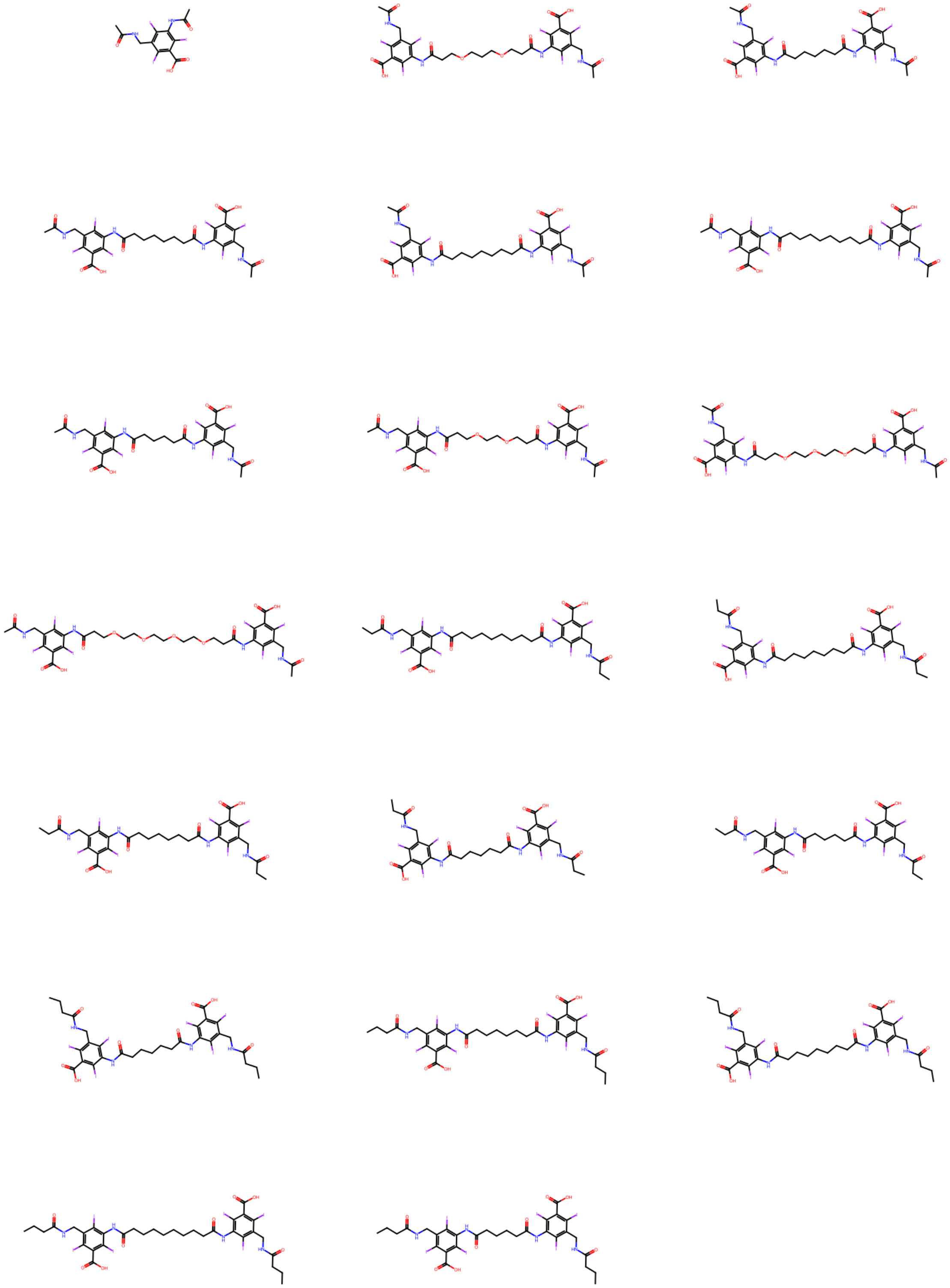

Figure S3. Molecular structures of compounds in cluster 3.

Cluster 4

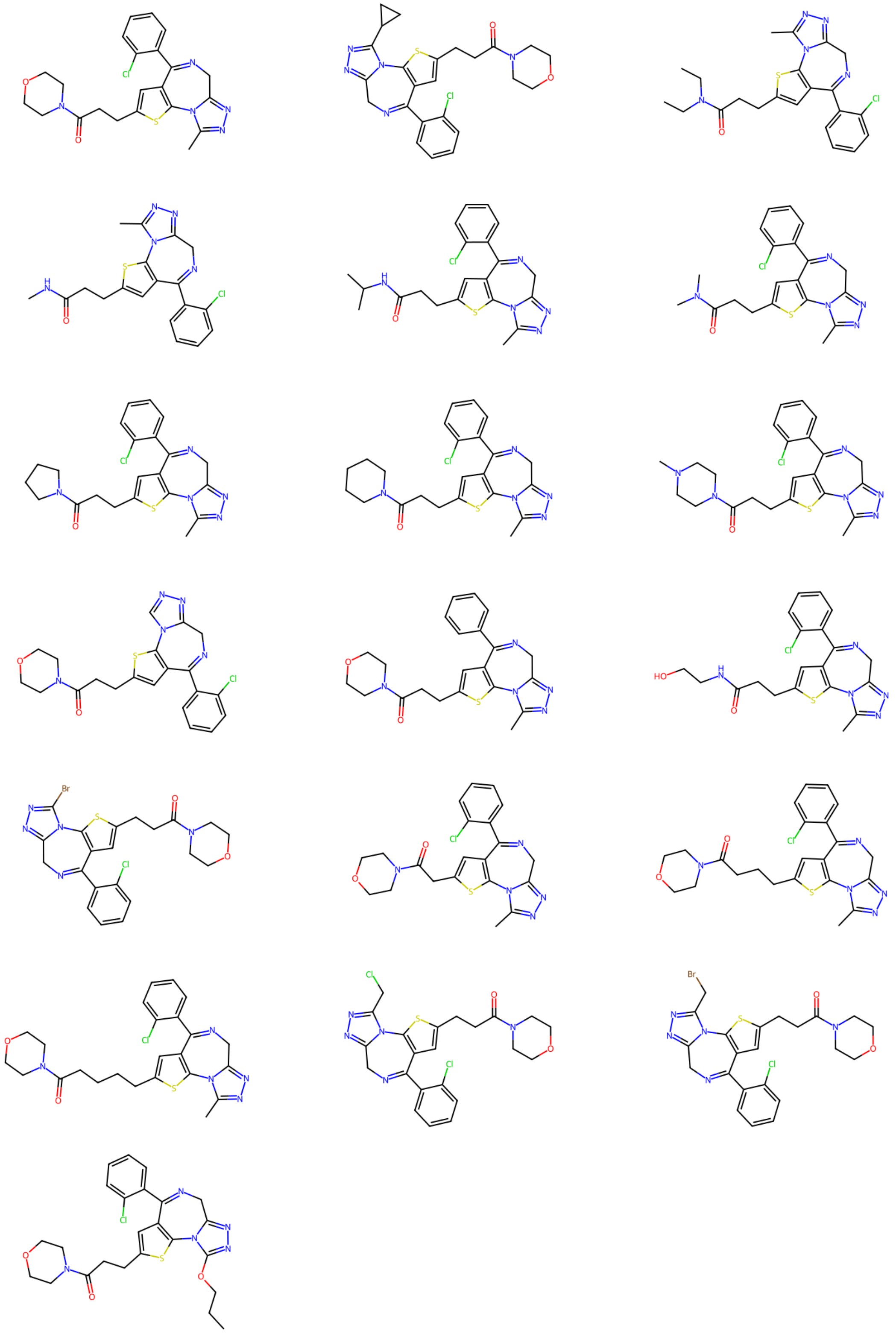

Figure S4. Molecular structures of compounds in cluster 4.

Cluster 5

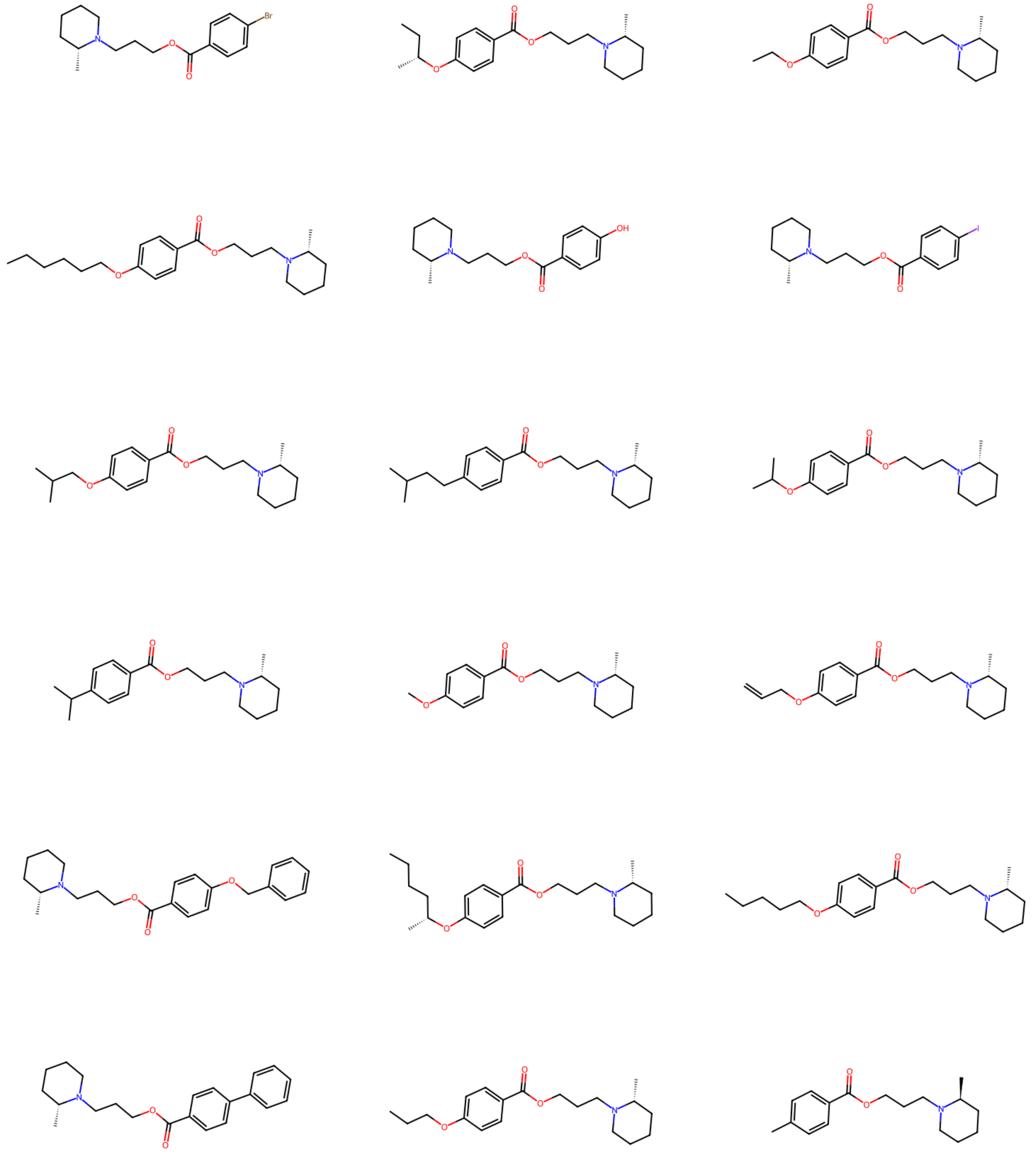

Figure S5. Molecular structures of compounds in cluster 5.

Cluster 6

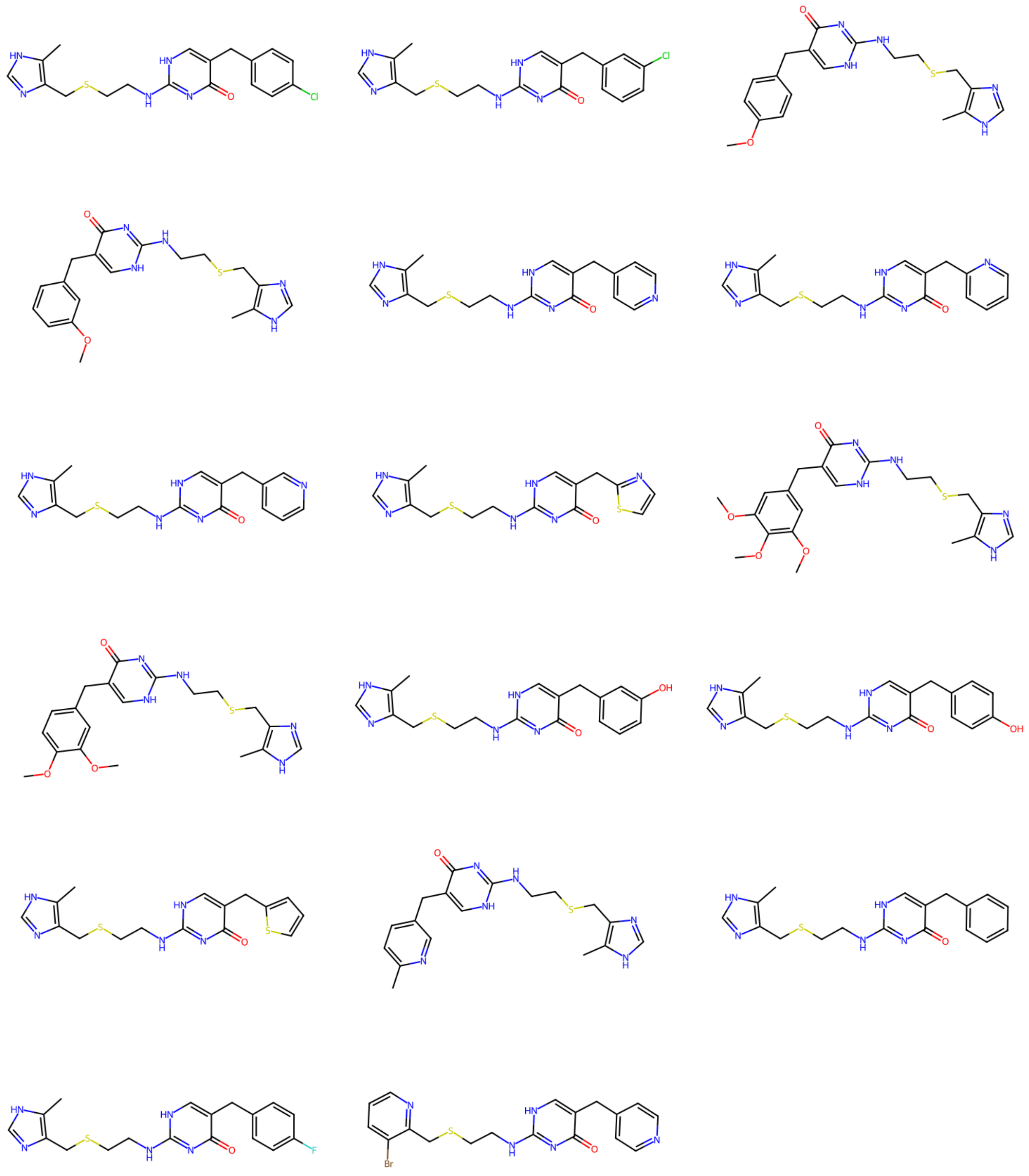

Figure S6. Molecular structures of compounds in cluster 6.

Cluster 7

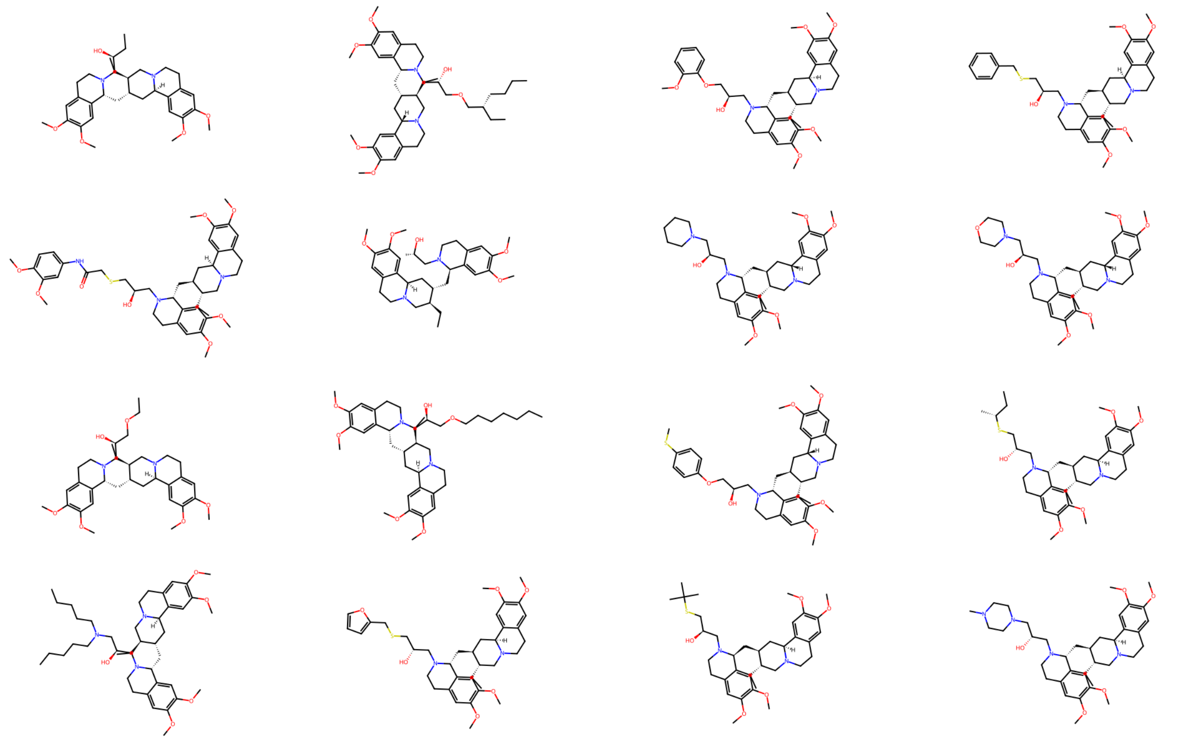

Figure S7. Molecular structures of compounds in cluster 7.

Cluster 8

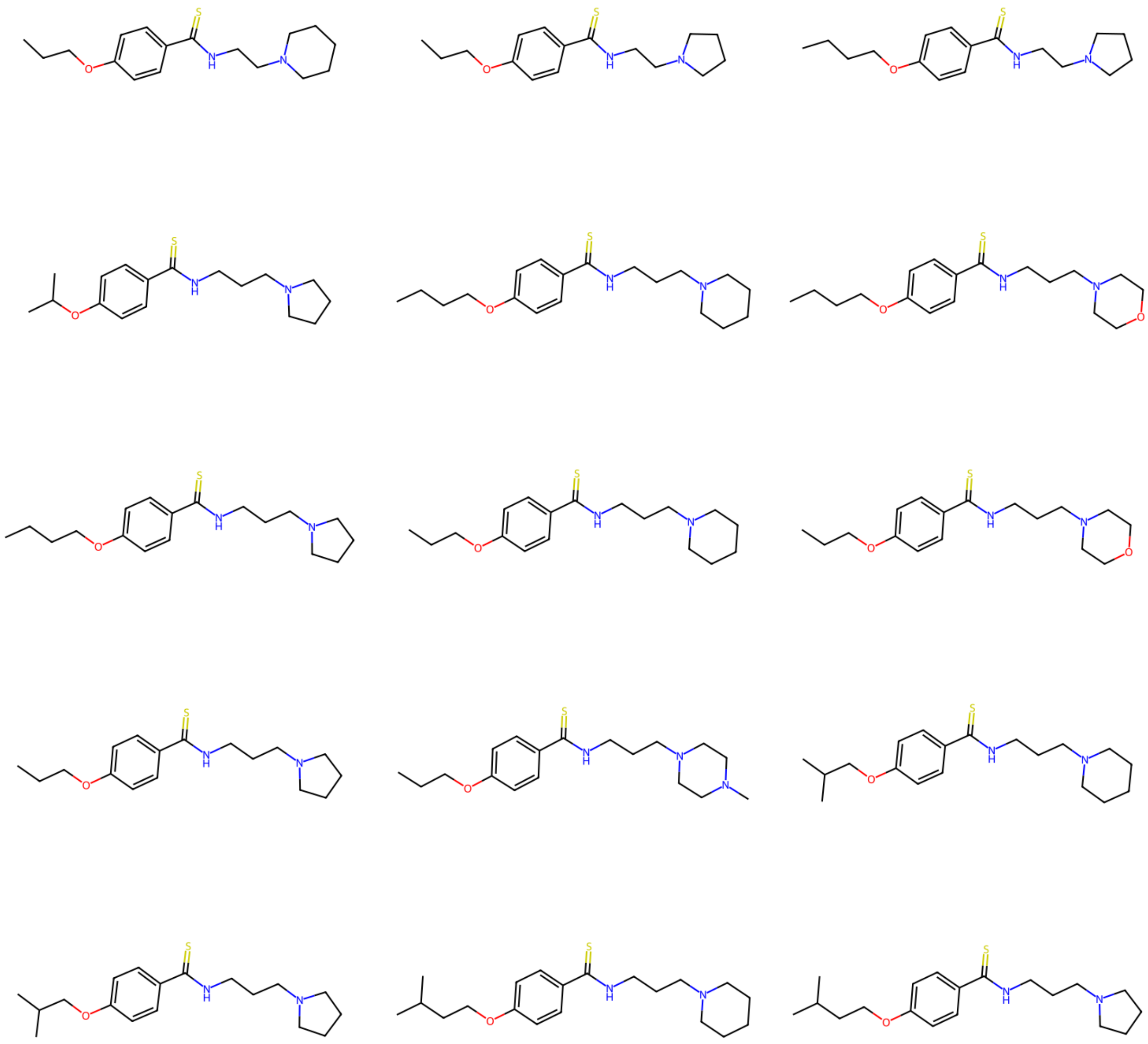

Figure S8. Molecular structures of compounds in cluster 8.

Cluster 9

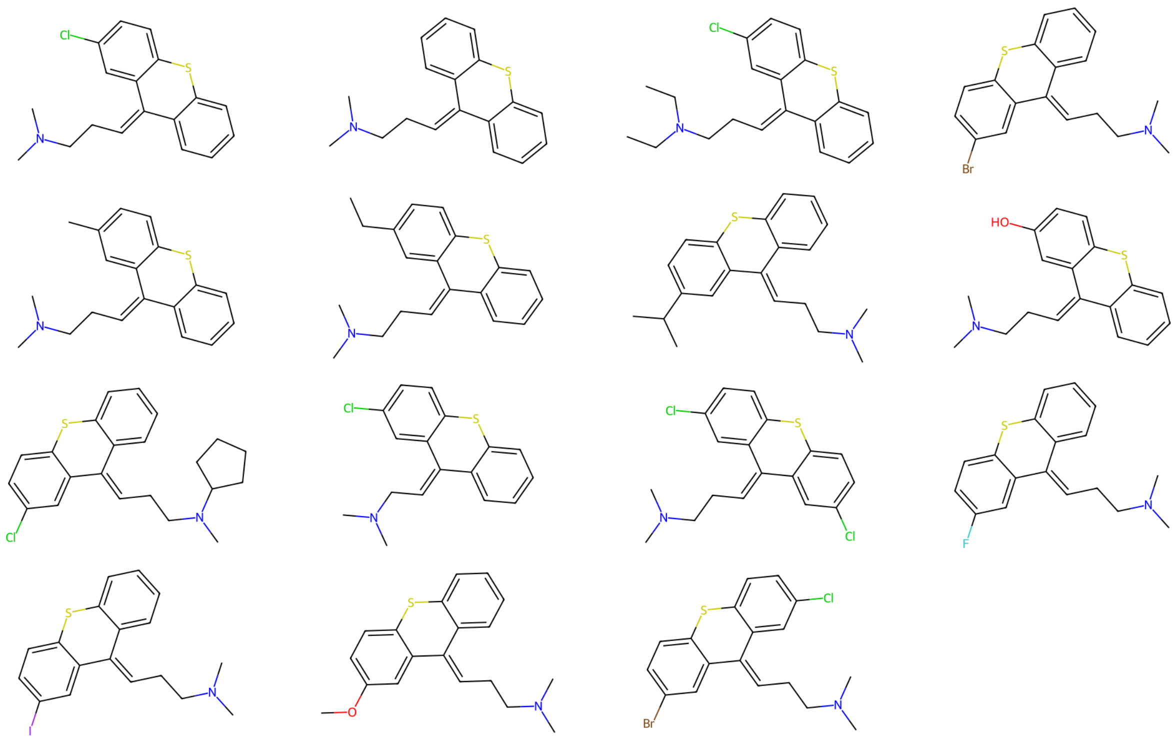

Figure S9. Molecular structures of compounds in cluster 9.

Cluster 10

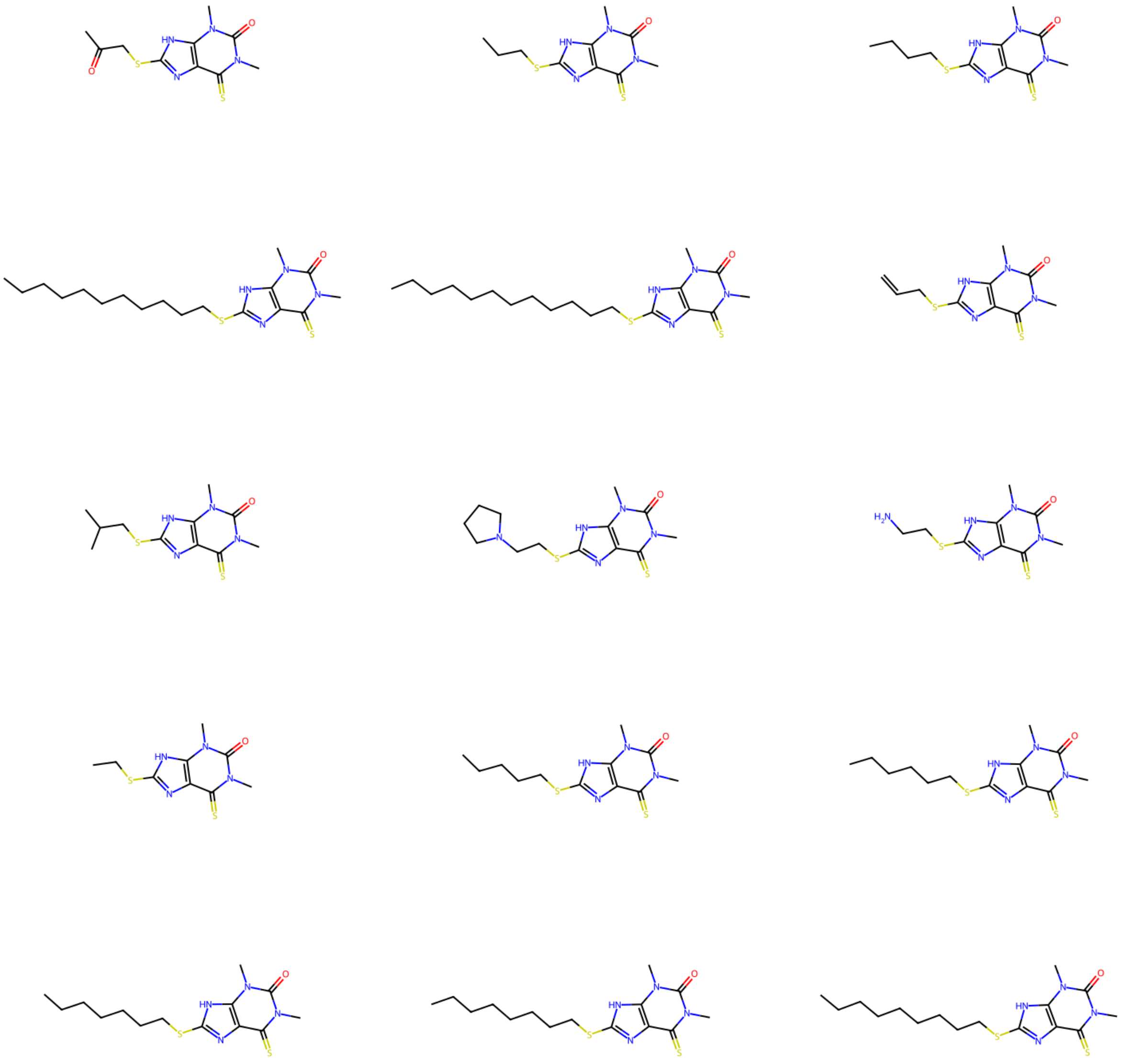

Figure S10. Molecular structures of compounds in cluster 10.

Cluster 11

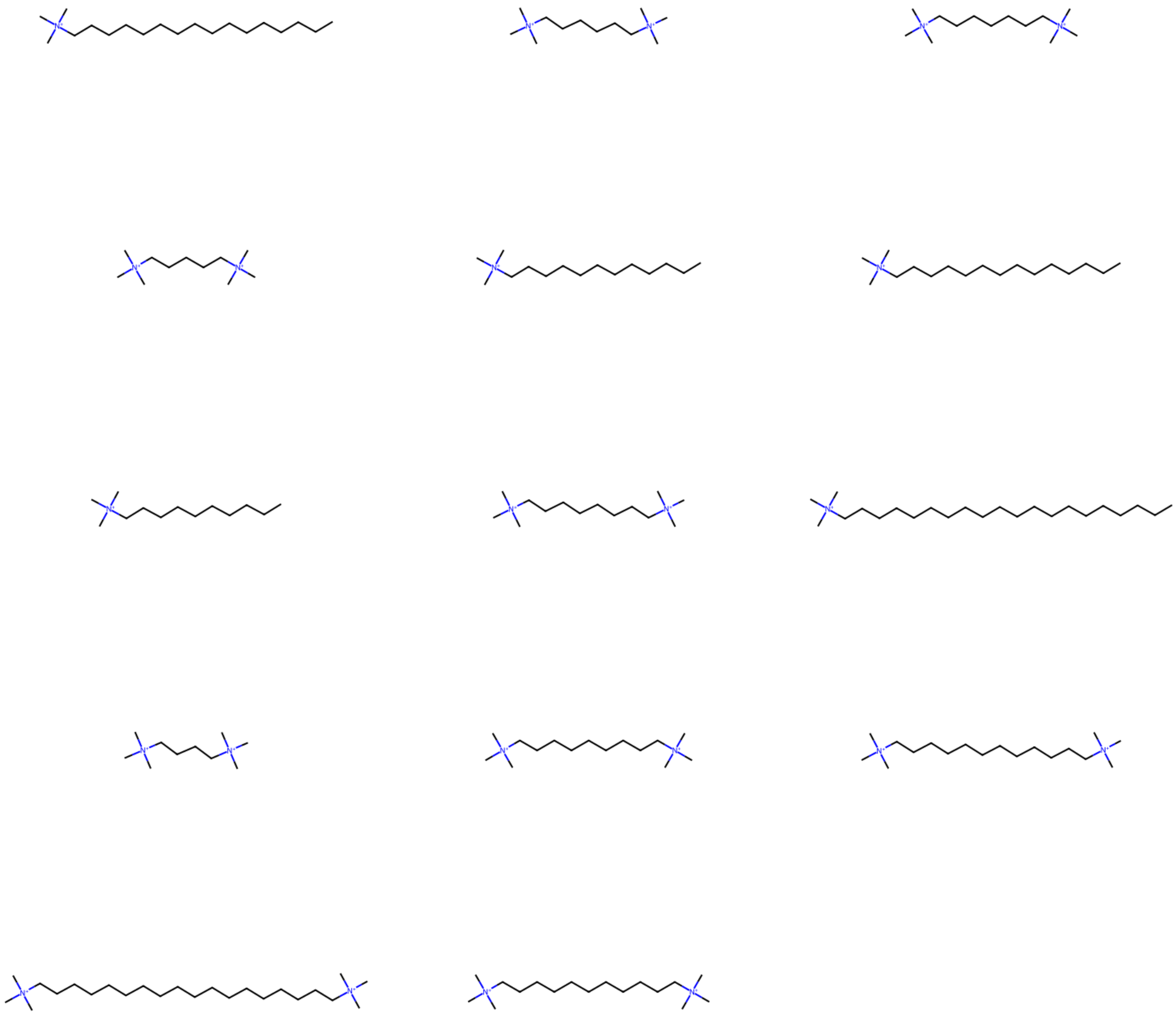

Figure S11. Molecular structures of compounds in cluster 11.

Cluster 12

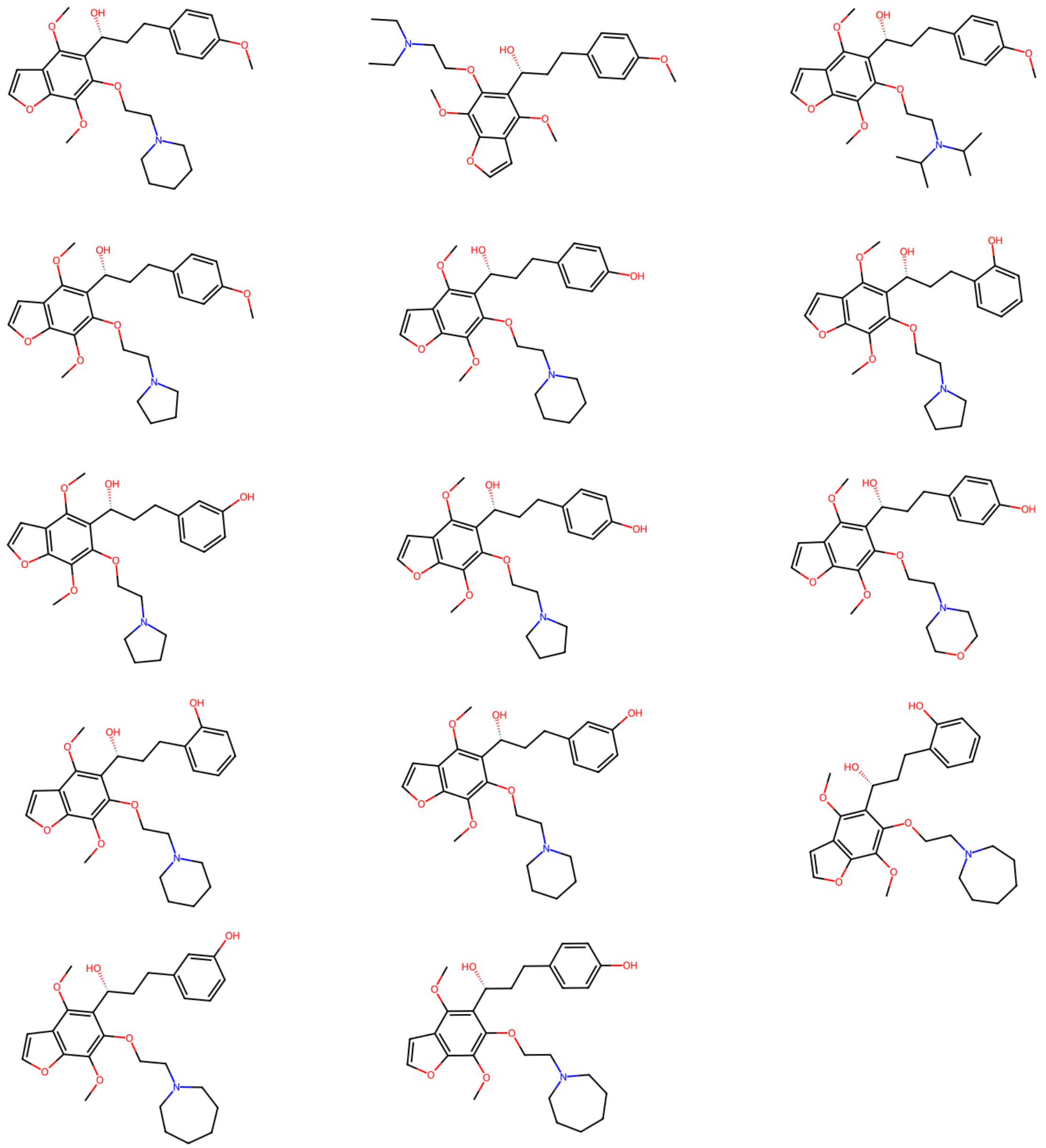

Figure S12. Molecular structures of compounds in cluster 12.

Cluster 13

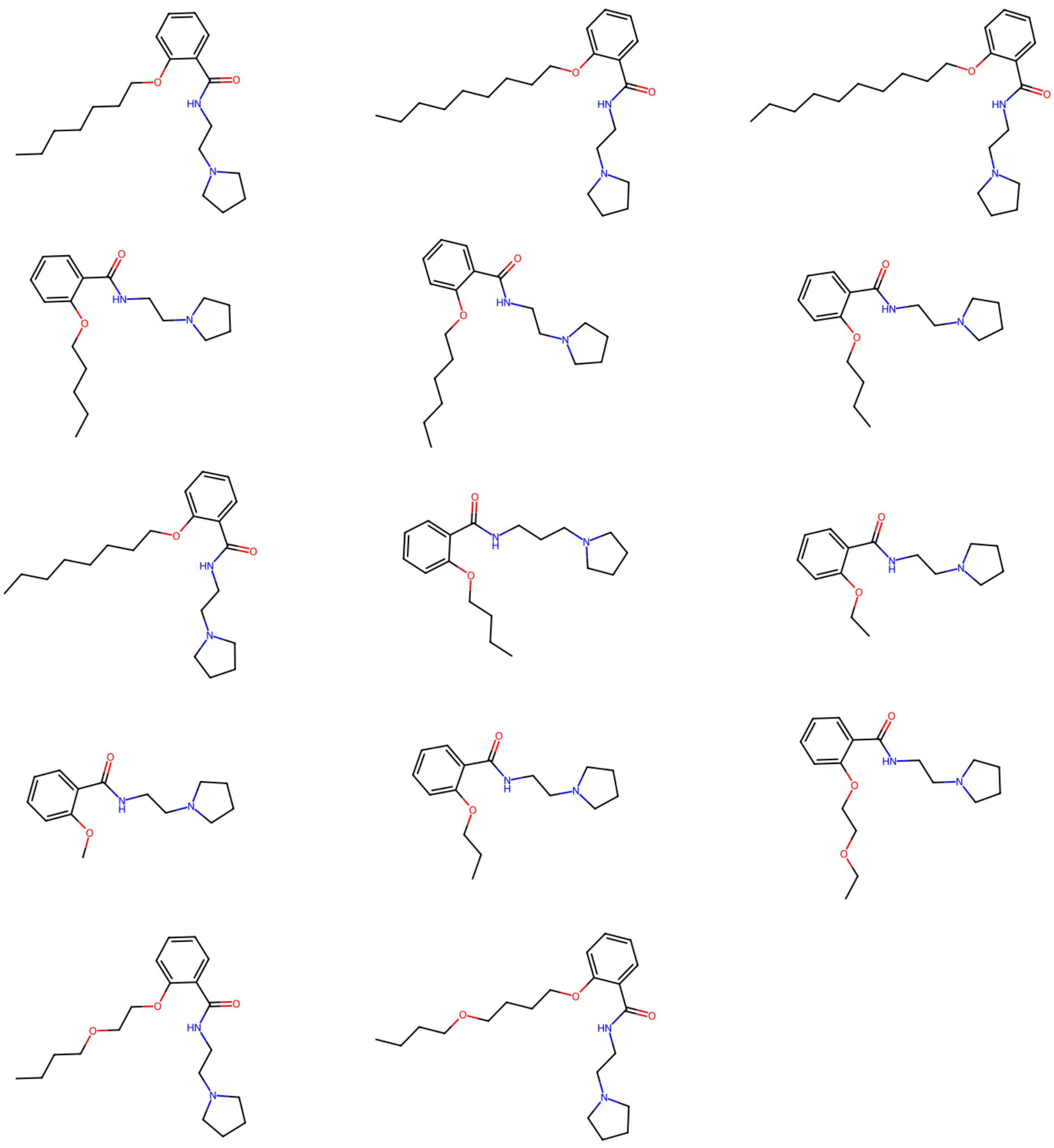

Figure S13. Molecular structures of compounds in cluster 13.

Cluster 14

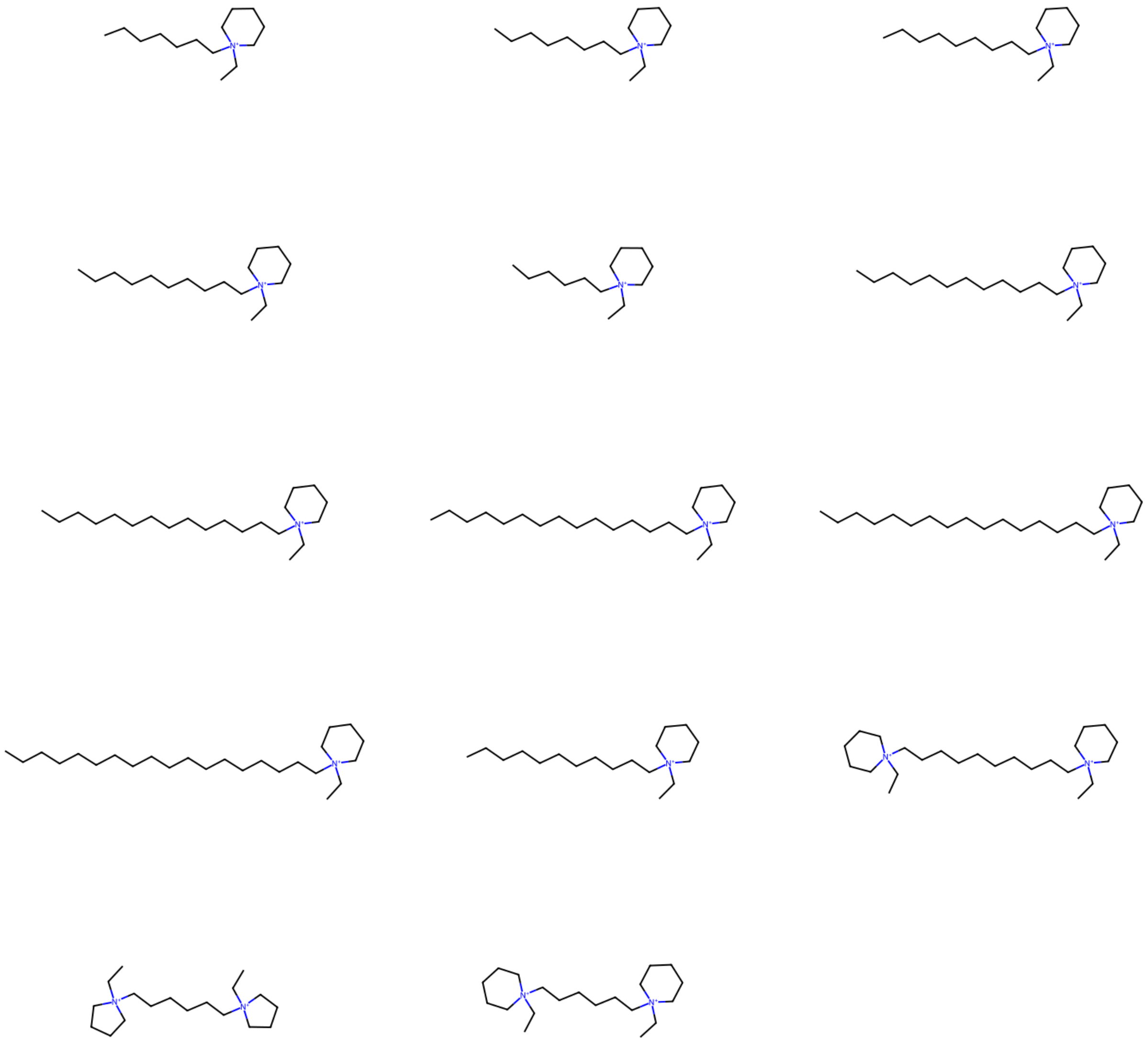

Figure S14. Molecular structures of compounds in cluster 14.

Cluster 15

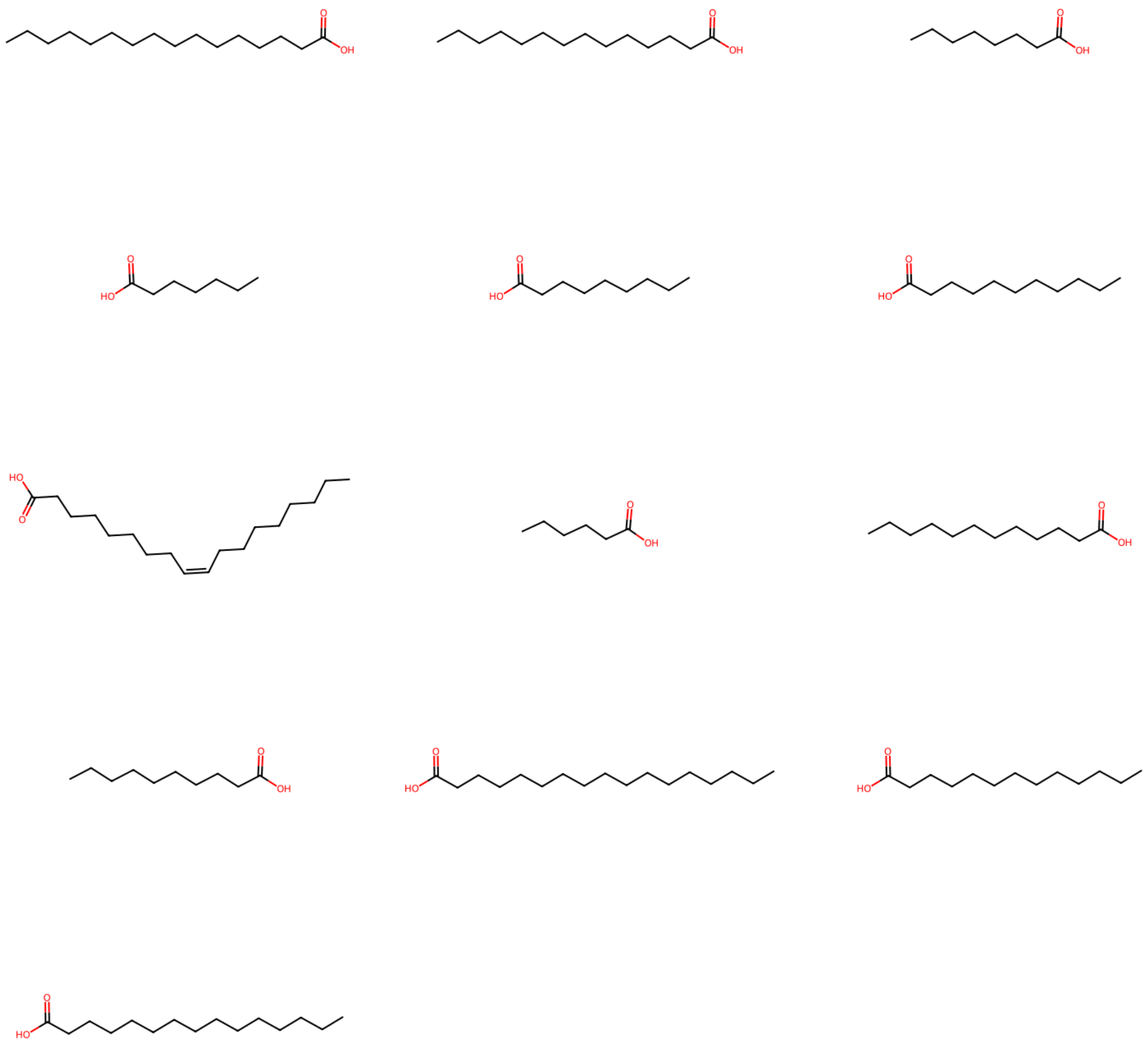

Figure S15. Molecular structures of compounds in cluster 15.

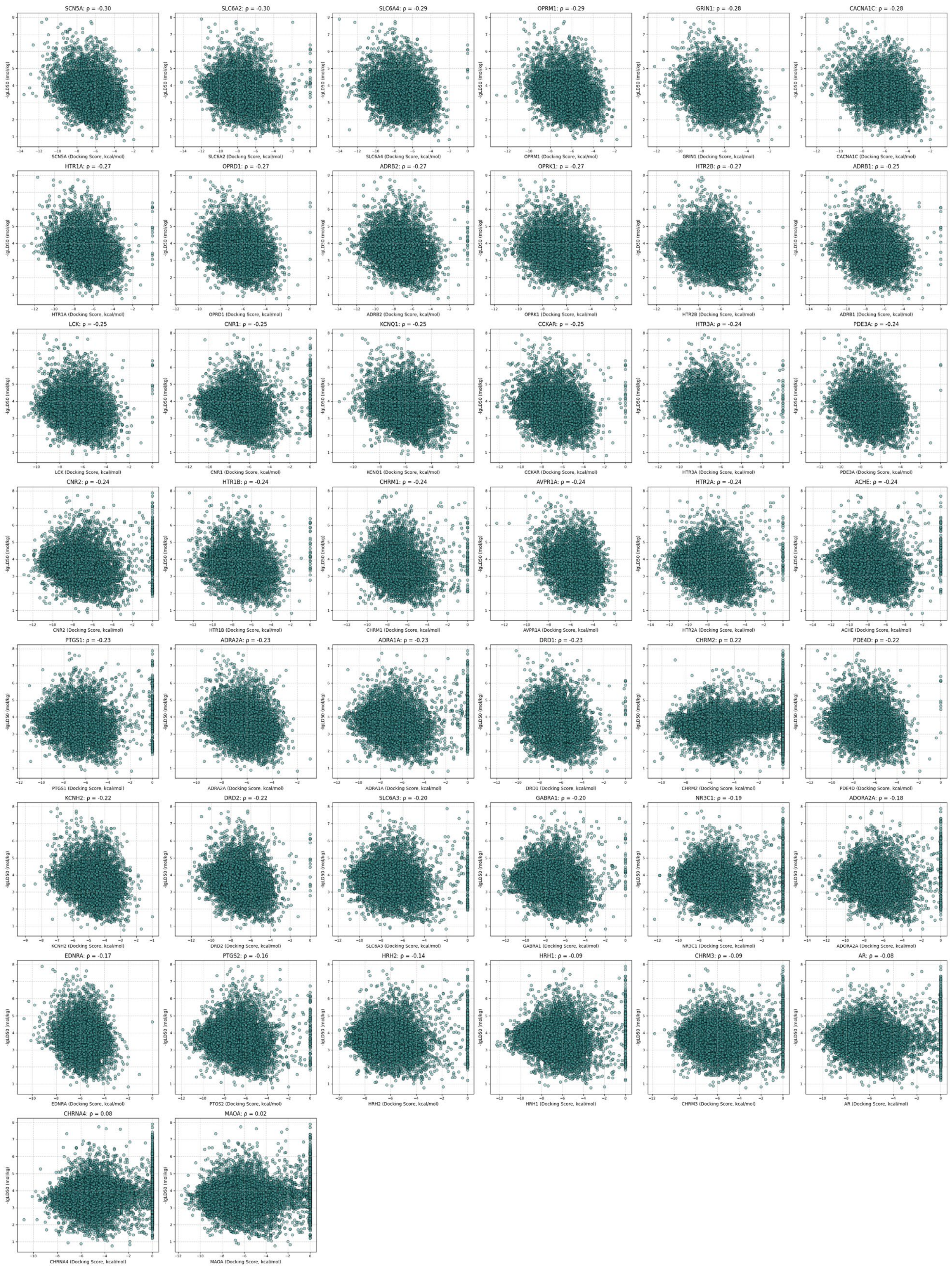

Figure S16. Scatter plots of docking scores versus pLD50 (mol/kg) for 12,654 ligands across

44 proteins, sorted by increasing p-value of Spearman's rank correlation. Each panel displays the protein identifier and the corresponding Spearman's correlation coefficient (ρ).
